# Influence of fermented feed additive on gut morphology, immune status, and microbiota in broilers

**DOI:** 10.1186/s12917-022-03322-4

**Published:** 2022-06-10

**Authors:** Wentong Peng, Mir Zulqarnain Talpur, Yuxian Zeng, Peipei Xie, Jincheng Li, Songbo Wang, Lina Wang, Xiaotong Zhu, Ping Gao, Qingyan Jiang, Gang Shu, Haijun Zhang

**Affiliations:** 1 Guangdong Laboratory of Lingnan Modern Agriculture, Guangdong Province Key Laboratory of Animal Nutritional Regulation and National Engineering Research Center for Breeding Swine Industry, College of Animal Science, South China Agricultural University, Guangzhou, China; 2 Key Laboratory of Feed Biotechnology of Ministry of Agriculture and Rural Affairs, Feed Research Institute, Chinese Academy of Agricultural Sciences, Beijing, China

**Keywords:** Fermented feed, Broiler, Microbiota, Small intestine histology, Intestinal immunity

## Abstract

**Background:**

This study examined the effects of a solid-state fermented feed additive (FFA) on the small intestine histology/morphology, immunity and microbiota of broilers. Two hundred eighty-eight day-old Arbor Acre chicks, were randomly assigned to one of four groups (each group has 6 replicates, with each replicate containing 12 chickens). The negative control (NC; basal diet), the positive control (PC; basal diet +antibiotic 15 ppm), the fermented feed additive low dose (FFL; basal diet + 0.3 kg/t FFA), and the fermented feed additive high dose (FFH; 3 kg/t FFA) with *Lactobacillus casei* (*L.casei*).

**Results:**

The study found that the FFH and FFL groups gained more weight (1-21d) and the FFL and PC diets had better feed conversion ratio (*P* < 0.05) than the NC from 0-42d. The FFH group had higher villus height (*P* < 0.05) in the duodenum than the PC and villus height to crypt depth ratio VH/CD compared to PC and FFL groups. The FFL chickens had greater (*P* < 0.05) jejunal and ileal villus height than PC and NC groups respectively. The FFL group had a higher ileal VH/CD ratio (*P* < 0.05). Jejunum VH/CD was higher in FFL and FFH (*P* < 0.05) than PC (*P* < 0.05). FFH had a smaller thymus than NC (*P* < 0.05). FFA diets also increased IL-10 expression (*P* < 0.05). While IL-1 and TLR4 mRNA expression decreased (*P* < 0.05) compared to NC. The microbiota analysis showed that the microorganisms that have pathogenic properties such as phylum *Delsulfobacterota* and class *Desulfovibriona* and *Negativicutes* was also significantly reduced in the group treated with FFH and PC while microorganisms having beneficial properties like *Lactobacillaceae* family, *Lactobacillus aviarus* genus and *Lactobacillus spp* were also tended to increase in the FFH and FFL fermented feed groups compared to the PC and NC groups.

**Conclusion:**

These findings suggested that the FFA diet may modulate cecal microbiota by reducing pathogenic microorganisms such as phylum *Delsulfobacterota* and class *Desulfovibriona* and *Negativicutes* improve beneficial microorganisms like *Lactobacillaceae* family, *Lactobacillus aviarus* genus and *Lactobacillus* spp. While FFA diet also affect immunity, and gene expression related to immunity.

**Supplementary Information:**

The online version contains supplementary material available at 10.1186/s12917-022-03322-4.

## Background

Up to 70% of the total cost of commercial poultry production is spent on the production of feed for broilers. Poultry farmers are turning to alternative or unconventional feed ingredients as a result of rising global feed prices. Unconventional feed ingredients may contain antinutritional factors (ANFs) that reduce feed digestibility, so this move is constrained by those ANFs. The results of previous studies have shown that fermentation raises crude protein levels, but it also lowers crude fiber levels [1], a number of ANFs and toxic compounds found in feed ingredients are also reduced [2]. In addition to improved nutritional properties, fermentation is linked to a high number of lactic acid bacteria (LAB), a low pH, and an increased concentration of organic acids [3]. Research shows that when these latter attributes are applied in combination or used alone, the feed is protected from pathogen contamination before it is fed [4], a benefit to the digestive health of the chicken [5, 6] as well as the development of chickens [7]. Fermented feed has long been used in pig nutrition [3], but now there is growing interest in using it in broiler rations to benefit gut health and production parameters [1].

Previously, fermentation was linked to improving the nutritional value of unusual broiler feed ingredients. In recent decades, the fermentation process has been used to create functional feeds with the potential to improve broiler GIT histology/morphology, immune status, and microbiota status. Following the ban on antibiotic growth promoters (AGP), there was an increase in demand for growth promoting agents other than AGP to meet the increased demand for antibiotic-free poultry [8]. Several AGP alternatives have been introduced to the market, including degrading enzymes, yeast extracts, plant extracts, fermented feed, prebiotics, symbiotics, and probiotics [9–11]**.** Probiotics and fermented feed have been evaluated as technically viable alternatives to antibiotics in broiler feed. Fermented feeds have been shown to promote growth [12], and improve feed palatability, as well as have a positive impact on nutrient utilization and reduce greenhouse gas (GHG) emissions [13].

Solid state fermentation (SSF) is being used in food processing and the manufacturing of traditional fermented foods in the Orient and Asia for centuries, and it is still in use today. SSF is used in the production of Japanese rice wine, in addition to fermented soybeans (sake) [14]. SSF is a type of microbial fermentation that takes place in the absence or near absence of free water; this is because it simulates the natural environment to which the selected microorganisms have naturally adapted. Due to perceived advantages in the production of various secondary metabolites and innovative food products, the use and development of SSF technology has increased dramatically in recent years, SSF is now used in the production of broiler diets such as sour cherry kernel through solid-state fermentation improved the nutritional composition of broiler diets [15–17]. Use of fungal SSF bioproducts in commercial broiler feed has also been found to positively influence fatty acid profile [18]. The gut microbiota has been linked to nutrient absorption, digestibility, and metabolism, and thus microbial composition and diversity influence animal productivity; additionally, diet, including feed components and feed additives, can modulate gut microbial composition and diversity [19]. The gut microbiota was also positively affected by fermented feed [20, 21], such as, hens fed a diet supplemented with Lactobacillus as starter cultures have a lower number of Enterobacteriaceae in their faeces [22], while fermented moist feed significantly decreased the number of Coliforms and Streptococci in broilers’ small intestine [5]. Numerous studies have demonstrated that *L. casei* strains can alter the GIT microbiota and thus the host’s immune response [10, 11]. Recently, researchers discovered that feeding broilers an SSF containing *Lactobacillus salivarius* increased crude protein content while decreasing glucosinolates [23]. *Lactobacillus plantarum* was found to increase caecal populations of Lactobacilli and Bifidobacteria spp. while decreasing caecal *E. coli* and Salmonella spp. in the diet of yellow feather broilers [24].

However, the broiler GIT microbiota contains hundreds of bacterial species, which makes traditional culture-based approaches difficult to cultivate and study intestinal microbiota composition and structure. Recently, the 16 s rDNA gene sequence has been used in a variety of studies to investigate microbial composition and diversity as well as their correlation to broiler growth performance [25] and immunity of animals [10]. However, it’s still unclear if FFA strengthens broiler immunity or changes the composition of their gut microbiota. The purpose of the study was to determine what effect FFA had on immune parameters, the expression of gut immunity-related genes, and the composition of the gut microbiota.

On the basis of the evidence presented above, we propose that adding fermented feed additives (FFA) to broiler chicken feed may improve gut immunity and the composition of gut bacteria without affecting the animals’ overall growth.

## Results

### Growth performance

The FFH and FFL groups gained weight faster than the NC group during the first 21 days, but there were no significant differences in body weight, FCR, ADG, or ADFI between the four groups (*P* > 0.05; Table 1). There were no significant changes in growth performance indices observed during the finishing stage (22–42 day). The FFL and PC groups have improved and have lower FCR than the NC group, but the FFH group has a lower FCR from 0 to 42 than the NC group (*P* < 0.05). Over the entire period, no other growth performance indices show a significant difference (0 to 42 days). Mortality was not significant at any time point, but was higher in the FFL group than in the NC and PC groups, with no mortality found in the FFH group from 0 to 21 days (*P* > 0.05 Table 1), lower mortality was observed due to treatment effects during the 21 to 42 day period and the entire period of 0 to 42 day.

**Table 1:** Effect of fermented feed supplementation and antibiotic on growth performance in broiler chicks^1^

Items^2^	NC	PC	FFL	FFH	*P*-value
1-21 day					
ADG, g	40.53 ± 0.70	41.69 ± 2.43	41.48 ± 2.51	42.87 ± 1.37	0.32
ADFI, g	48.31 ± 2.60	48.42 ± 2.44	48.85 ± 1.88	50.49 ± 1.53	0.38
FCR (g:g)	1.19 ± 0.05	1.16 ± 0.01	1.179 ± 0.03	1.178 ± 0.30	0.62
Mortality^3^	0.19 ± 1.19	1.19 ± 1.19	3.33 ± 2.04	0	0.16
21 d BW	904.90 ± 19.94^b^	931.66 ± 53.69^ab^	971.02 ± 36.42^a^	972.16 ± 24.75^a^	0.02
22–42 d					
ADG, g	76.90 ± 15.98	71.01 ± 6.61	77.28 ± 16.94	80.55 ± 9.97	0.71
ADFI, g	140.42 ± 23.92	131.68 ± 13.59	142.60 ± 24.86	148.85 ± 13.33	0.59
FCR (g:g)	1.840 ± 0.090	1.853 ± 0.020	1.863 ± 0.110	1.855 ± 0.089	0.98
Mortality^3^	8.44 ± 2.57	4.87 ± 1.72	1.82 ± 1.82	3.33 ± 2.04	0.34
42 d BW	2891.27 ± 376.0	2762.44 ± 209.0	2863.69 ± 435.3	3058.68 ± 203.9	0.55
42 d					
ADG, g	58.04 ± 7.87	56.61 ± 6.06	58.46 ± 8.95	60.93 ± 4.79	0.81
ADFI, g	92.70 ± 11.58	88.64 ± 8.46	93.39 ± 12.61	97.69 ± 6.16	0.57
FCR (g:g)	1.612 ± 0.013^b^	1.567 ± 0.022 ^a^	1.590 ± 0.050 ^a^	1.605 ± 0.042 ^ab^	0.05
Mortality^3^	9.63 ± 2.88	6.06 ± 1.57	5.15 ± 3.47	3.33 ± 2.04	0.63

^1^n = 6 replicates per treatment

^2^Negative control (NC), Positive control (Antibiotic) (PC), Fermented feed (low dose) (FFL), Fermented feed (high dose) (FFH)

^3^Values transformed by arc sine (angular) transformation

*ADG* average daily gain, *ADFI* average daily feed intake, FCR (feed: gain = g: g), feed conversion ratio, *BW* body weight

*Note*: Values with different superscripts ^a^ ^b^ in the same row differ significantly (*P <* 0.05)

### Slaughter performance and immune organs index

**Fig. 1:**
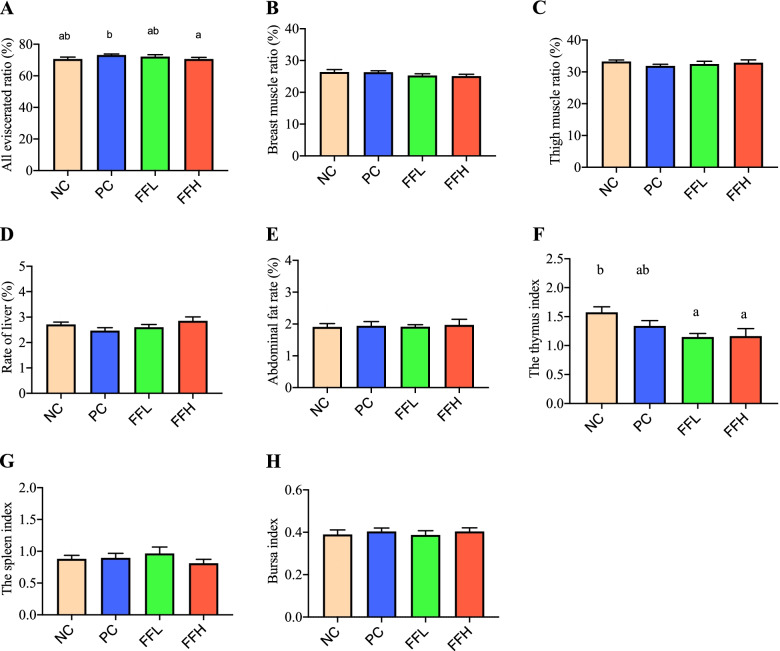
Effect of antibiotic and fermented feed on eviscerated yield, component parts, meat ratios, and immune organs in broiler chicken. Negative control (NC), Positive control (Antibiotic) (PC), Fermented feed (low dose) (FFL), Fermented feed (high dose) (FFH). Values with different superscripts in the same row differ (*P <* 0.05). Different color bar plots showing different groups. *n* = 6 replicates per treatment

All eviscerated ratios, breast and thigh muscle ratios, and the relative rate of liver and fat were all evaluated, and the PC group had a higher all eviscerated ratio than the FFH group (*P* < 0.05 Fig. 1A). However, no differences in breast muscle, thigh muscle, liver, or abdominal fat rate parameters were discovered (*P* > 0.05 Fig. 1B, C, D, E).

Spleen, bursa, and thymus index were also measured as immune organs. The NC group had a higher thymus weight than the FFL and FFH groups (*P* < 0.05 Fig. 1F), but the spleen and bursa were not different (Fig. 1G, H).

### Duodenum, jejunum, and ileum morphology

**Fig. 2:**
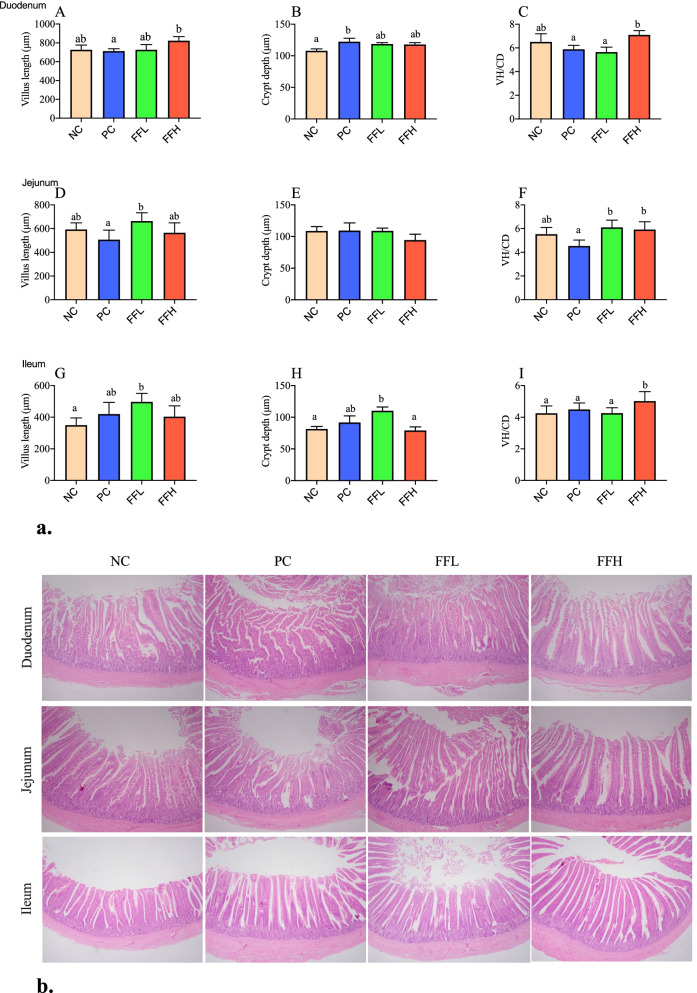
**a** Effect of antibiotic and fermented feed on duodenum, jejunum, and ileum in broiler chicken. Negative control (NC), Positive control (Antibiotic) (PC), Fermented feed (low dose) (FFL), Fermented feed (high dose) (FFH). Values with different superscripts in the same row differ (*P <* 0.05). Different color bar plots showing different groups. *n* = 6 replicates per treatment. **b** Effect of antibiotic and fermented feed on small intestine’s (duodenum, jejunum, and ileum) morphology in broiler chicken. Negative control (NC), Positive control (Antibiotic) (PC), Fermented feed (low dose) (FFL), Fermented feed (high dose) (FFH). The gross and microscopic views of different parts of chickens’ intestine in treatment groups and control group. The intestine parts duodenum, jejunum, and ileum sections were used. Histology was assessed for intestine sections embedded in paraffin – sections stained with hematoxylin and eosin. *n* = 6 chicken per treatment

The morphometric and histological characteristics of the chicken digestive tract in various groups are depicted in Fig. 2a and b. While diet supplementation with fermented feed had a significant effect on the morphology of the duodenum, jejunum, and ileum, the intestines of all groups exhibited normal tissue architecture. The chicken fed FFH had a greater (*P* < 0.05 Fig. 2a) villus height in the duodenum than the chicken fed PC, as well as a greater VH/CR ratio in the duodenum and ileum than the chicken fed PC and the FFL group (*P* < 0.05 Fig. 2a, C, I). The FFL chickens had significantly higher jejunal and ileal villus heights (*P* < 0.05 Fig. 2a, D, G) than the PC and NC groups. Nonetheless, jejunum VH/CD ratios were significantly higher (*P* < 0.05 Fig. 2a, F) in the FFL and FFH groups than in the PC group. However, the duodenal and ilea crypts were significantly larger in PC and FFL (*P* < 0.05 Fig. 2a, B, H), respectively, than in the NC group. While the jejunum crypts were not different between groups (Fig. 2a, E). Separate tall and arranged intestinal villi with free lumen were observed in duodenal segment sections from the FFH group (Fig. 2b). Apart from partially destroyed villi with some fused villi, FFL treatment resulted in an increase in the intestinal gland layer (Fig. 2b). The FFL group demonstrated increased enterocyte proliferation and goblet cell metaplasia, resulting in prominent fusion villi in the ileal segment.

### Pro-inflammatory cytokine

**Fig. 3:**
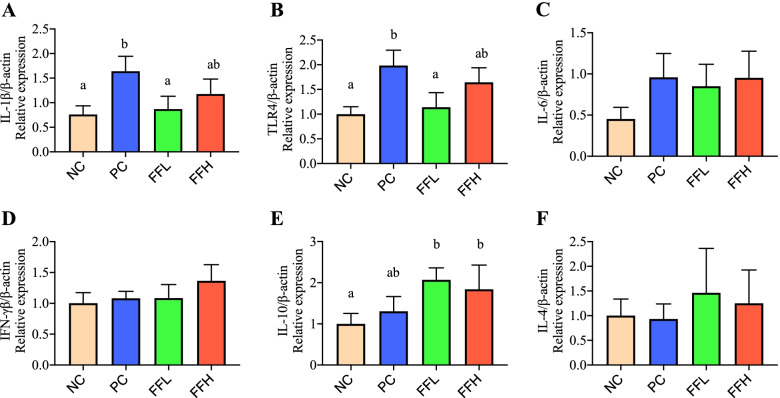
Effect of antibiotic and fermented feed on the relative gene expression of inflammatory cytokines of broiler chickens. Negative control (NC), Positive control (Antibiotic) (PC), Fermented feed (low dose) (FFL), Fermented feed (high dose) (FFH). Values with different superscripts in the same row differ (*P <* 0.05). Interleukin 1 beta, Toll-like receptor 4, Interleukin 6, Interleukin 10, Interleukin 4, Interferon gamma. n = 6 replicates per treatment

The PC group had significantly higher levels of pro-inflammatory cytokine IL-1 β and TLR4 gene expression than the NC group (*P* < 0.05; Fig. 3A, B), whereas IL-10/ β was significantly higher in the FFL group than the NC group (*P* < 0.05; Fig. 3E), although it was similar between the PC, FFL, and FFH groups, whereas IL-6/ β, IFN- γβ/β and IL-4/β were not significantly different between groups (Fig. 3C, D, F).

### Analyses of microbiota composition

**Fig. 4:**
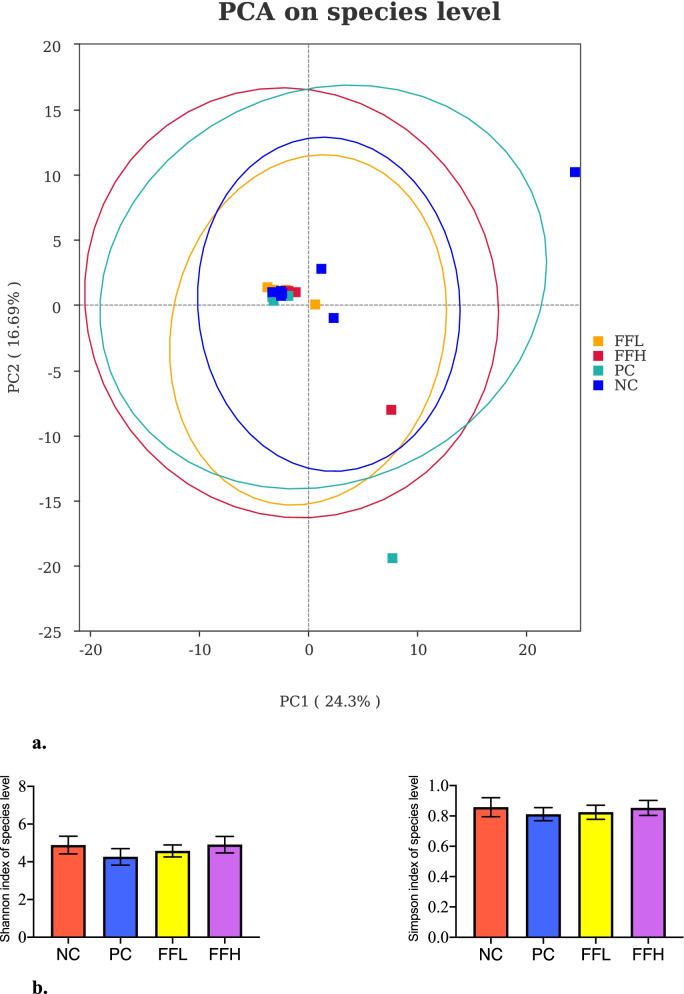
**a** PCA on species level. Negative control (NC), Positive control (Antibiotic) (PC), Fermented feed (low dose) (FFL), Fermented feed (high dose) (FFH). Principal component analysis scores are plotted based on the relative abundance of OTUs of gut microbiota. **b** Alpha diversity index Shannon and Simpson on species level. Negative control (NC), Positive control (Antibiotic) (PC), Fermented feed (low dose) (FFL), Fermented feed (high dose) (FFH). Principal component analysis scores are plotted based on the relative abundance of OTUs of gut microbiota

**Fig. 5:**
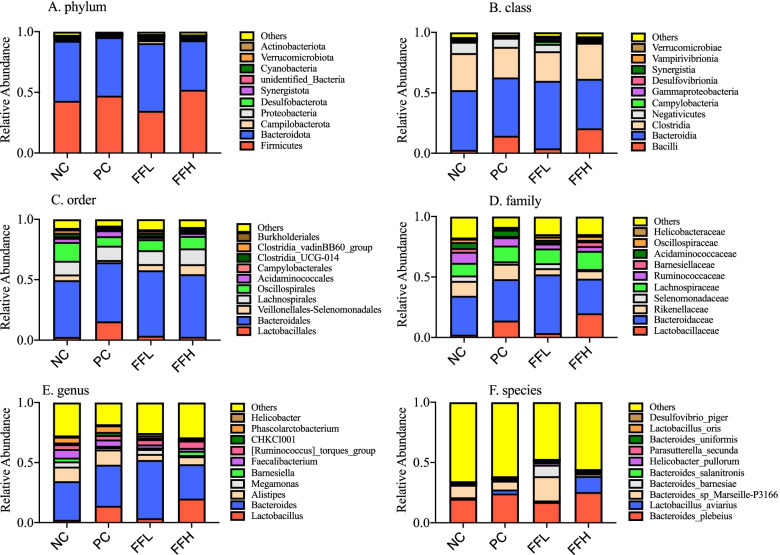
Effect of antibiotic and fermented feed on the Relative Abundance of broiler chickens. Negative control (NC), Positive control (Antibiotic) (PC), Fermented feed (low dose) (FFL), Fermented feed (high dose) (FFH). The microbiome compositions in cecum at phylum, class, family genus, and species-level composition of the caecal microbiome of chicken. A colorcoded bar plot shows the average bacterial phylum, class, family, genus, and species distribution in different treatment groups and control groups. Each bar chart represents the relative abundance of each group. Each color represents a specific bacteria phylum (**A**) a specific bacteria class (**B**) a specific bacteria order (**C**) a specific bacteria family (**D**) a specific bacteria genus (**E**) and a specific bacteria species (**F**)

**Fig. 6:**
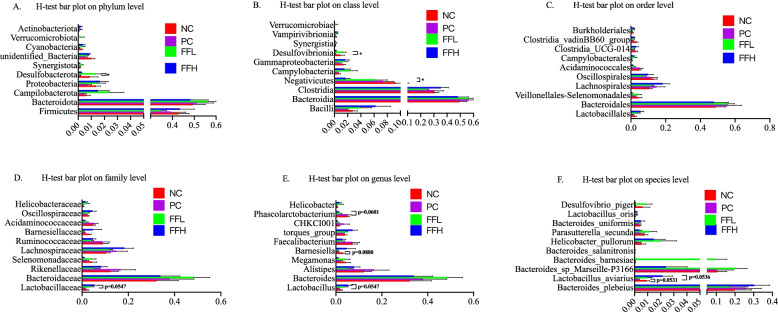
Effect of antibiotic and fermented feed on the H-test bar plot on phylum, class, order, family, genus, and species level of broiler chickens. Negative control (NC), Positive control (Antibiotic) (PC), Fermented feed (low dose) (FFL), Fermented feed (high dose) (FFH). The bacterial clades showing differences at phylum, class, order, family, genus, and species levels in different treatment groups and control groups. (**A**) bacterial clades significantly different at the phylum level in different groups (*P* < 0.05 is showing significant difference). (**B**) bacterial clades significantly different at the class level in different groups (*P* < 0.05 is showing significant difference). (**C**) bacterial clades significantly different at the order level in different groups (*P* < 0.05 is showing significant difference). (**D**) bacterial clades significantly different at the family level in different groups (*P* < 0.05 is showing significant difference). (**E**) bacterial clades significantly different at the genus level in different groups (*P* < 0.05 is showing significant difference). (**F**) bacterial clades significantly different at the species level in different groups (*P* < 0.05 is showing significant difference)

**Fig. 7:**
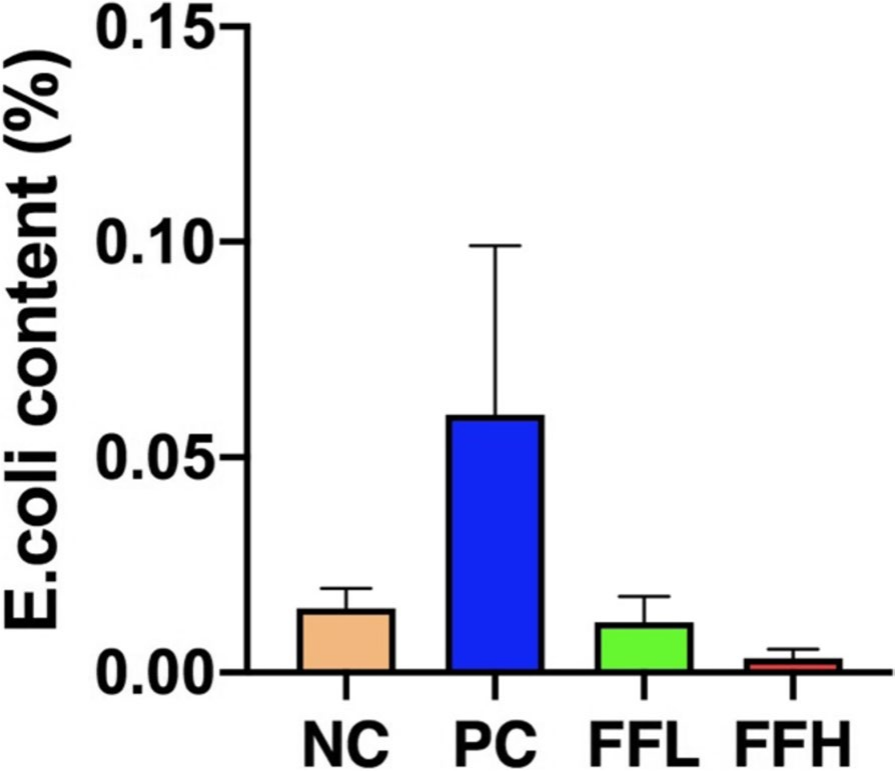
Effect of antibiotic and fermented feed on E.coli count in broiler chickens. Negative control (NC), Positive control (Antibiotic) (PC), Fermented feed (low dose) (FFL), Fermented feed (high dose) (FFH)

Afer fltering out low-quality and short sequence reads,there were 1,781,154 high quality sequence reads in total samples among all treatment groups. The median number of sequences in each sample was 68,506 (range from 51,107 to 87,680). Alpha Diversity is used to analyze the diversity of species within community samples. In general, sequences with greater than 97% identity are clustered into one operational taxonomic unit. Two-way ANOVA indicated no signifcant diferences among different groups in observed species, Shannon, Simpson, coverage ranged from 97 to 98%, indicating that the measurement depth has met the requirements (Fig. 4b). PCA analysis was used to visualize the differences in the caecal microbiota between the groups. PC1 explained for 33.06% of the variance, while PC2 explained for 26.87% of the variance (Fig. 4a). The PCA revealed that the cecal sample species were distinct between the groups and the control NC group. We observed that *Firmicutes*, *Bacteroides*, and *Proteobacteria* were the dominant phyla in each of the four groups (Fig. 5A). On the other hand, the dominant class consisted of *Bacilli*, *Bacterioda*, *Clostridia*, and *Negativicutes* (Fig. 5B). On the other hand, *Lactobacillales*, *Bacteroidales*, and *Veillonellales-Selenomonadales* were the dominant Order (Fig. 5C). Whereas *Lactobacillaceae*, *Bacteroidaceae*, and *Rikenellaceae* were the dominant families (Fig. 5D). Although the dominant genera were *Lactobacillus*, *Bacteroides*, and *Alistipes* (Fig. 5E). *Bacteroides*_*plebeius*, *Lactobacillus*_*aviarius*, and *Bacteroides*_*sp*_*Marseille_P3166* were the dominant species (Fig. 5F). No significant differences in the abundance of the major phyla *Firmicutes* and *Bacteroides* were observed between groups, but the FFH and PC groups had a lower abundance of the *Delsulfobacterota* phylum than the FFL and NC groups (*P* < 0.05; Fig. 6A). *Desulfovibriona* was also significantly less abundant in the group treated with FFH and PC compared to the FFL and NC groups (*P* < 0.05; Fig. 6B), whereas *Negativicutes*, a gram-negative bacterium belonging to the phylum *Firmicutes*, was significantly less abundant in the FFH group compared to the other groups (*P* < 0.05; Fig. 6B). The *Lactobacillaceae* family, which contains well-known probiotic bacteria, was tended to increase in the FFH and FFL fermented feed groups compared to the PC and NC groups (*P* > 0.05; Fig. 6D). Fermented feed groups FFH and FFL also tended to increase the abundance of *Lactobacillus* spp. (*P* < 0.05; Fig. 6E). The FFH group increased the abundance of *Lactobacillus aviarus*, followed by the FFL group but not differ significantly (*P* > 0.05; Fig. 6F). While order level was not different between groups (Fig. 6C). In comparison to the control NC group, the PC group had a lower abundance of *Lactobacillus aviarus* species (*P* > 0.05; Fig. 6F). Furthermore, the genus *E. coli* caused the majority of diarrhea in birds when they were fed fermented diets, but not when they were fed the positive control diet or the control diet (Fig. 7). *E. coli* levels were lowest in birds fed fermented diets with a high dose.

## Discussion

Microbial fermentation was recently developed as a low-cost method of increasing the nutritional value of broiler feed. The demand for fermented feed has increased as a result of the benefits of GIT health and growth performance for the broiler industry’s use of the fermenting process [26, 27]. Numerous studies have demonstrated that fermentation of feedstuffs with *Lactobacillus* can improve chicken growth performance [26, 27], though some studies have found that Lactobacillus fermentation of feedstuff has no effect on growth performance [2]. On the 21st day of this study, the fermented feeds FFL and FFH groups gained more weight than the PC group, and at the end of the experiment, the PC and FFL group had a significantly lower FCR. As demonstrated by Skrede et al. [26] *Lactobacillus* fermentation was found to have a positive effect on weight gain and feed conversion in wheat and barley diets. Improved feed conversion suggested that fermentation had a positive effect on nutrient digestibility and energy utilisation, which was confirmed in other studies [28]. Improved growth efficiency may be attributed to the development of a well-balanced microflora provided by fermented feed in the broiler diet. Proper fermented feed supplementation can foster the colonization of beneficial microflora in the intestines, resulting in increased broiler growth efficiency [29].

Better digestion and absorption in birds may be attributed to fermentation feeds, which in turn improves production output of birds [30]. The function of intestinal villi is strongly linked to increased villus height [31]. In our research, FFH-fed chickens had higher villus height and VH/CR in the duodenum and ileum (*P* < 0.05). The FFL chickens had higher jejunal and ileal villus height (*P* < 0.05) and a higher ileal VH/CD (*P* < 0.05). In both the FFL and FFH groups, however, jejunum VH/CD was higher (*P* < 0.05). Previous research has shown that fermented-product diets improve the structure and function of the small intestine in poultry [32, 33] as a result of which performance parameters have improved [30, 34].

Fermented feed can improve histology by inhibiting excessive inflammation in the GIT, according to previous studies of Missotten et al. [5], a recent research suggests that feeding chickens lactic acid-fermented diets, which are typically higher in Lactic acid bacteria (LAB), increases their resistance to infectious disease. Although the precise immunomodulatory effects of LAB in broilers are unknown, they may stimulate the production of cytokines by various subsets of immune cells, which play critical roles in the induction and regulation of immune responses. There is some evidence that some strains of *Lactobacilli* down-regulate TLR4 expression [35]. IL-10 is generally known to be an anti-inflammatory cytokine, IL-10 production and regulatory T-cells are involved in the immune tolerance to intestinal microbiota [36, 37]. In our study IL-1β, TLR4, and IL-6 expressions were decreased and IL10 expressions were increased with fermented feed FFL and FFH. This is in agreement with our finding that dietary fermented soybean meal in piglets decreased IL-1β concentrations and increased IL-10 concentration than their corresponding controls [38]. To determine the immune system of animals, not only the relative weight of lymphoid organs but other parameters of immunity status also need to consider [39]. Lowest weight may not necessarily be linked with lower production of lymphoid cells [40, 41];. In the present study thymus index of the NC group was significantly higher than the FFL and FFH group while no significant differences were found between different groups in spleen and bursa weight. Similarly, Tang et al. [42] and Choi et al. [43] found no significant differences in the relative weights of the spleen, thymus, and bursa of Fabricius in broilers after feeding with fermented feed. The above findings suggest that lymphoid compartments vary in their responses to the inclusion of fermented feed in chicken due to the different roles in the immune system. The bursa of Fabricius and spleen are sites of differentiation of B cells and T cells, respectively [44]. Variations in thymus weight could be associated with changes in the lymphoid organs’ function. Hence higher thymus index in the NC group might be due to higher exposure of infections or a reduced capacity to maintaining production potential to meet the sanitation challenges [45].

The microbiota has a significant impact on the host immune system. Immune system development, organismal health, and disease have all been linked to the gut microbiota [46], The lower thymus weight was found in fermented feed, it is important to realise that the lowest weight may not necessarily be linked with lower production of lymphoid cells; so this is essential that also consider other parameters of immunity status [40] and changes in the gut microbiota during early life can have a significant impact on the host immune system and these effects last for a long time [47]. When compared to conventional animals, germ-free animals had significantly lower thymus weight and development [48], however, in order to better understand how the thymus and gastrointestinal system interact, more research is needed.

The PCA revealed that the PC, FFL and FFH groups were correlated with each other but presented a different state from control group. Diets have an effect on the gut bacterial community and the abundance of bacterial metabolites; consequently, the composition of the gut microbiota has an effect on nutrient digestibility [49, 50]. The *Desulfobacterota* phylum (formerly *Deltaproteobacteria*) is significantly enriched in intestinal inflammation and injury in infected chickens’ guts [51, 52], whereas the abundance of class *Negativicutes*, a gram-negative bacterium belonging to the phylum *Firmicutes*, was significantly lower in the FFH group than in the other groups. The members of the *Negativicutes* family have a gram-negative cell wall composition [53]. Lower abundance in FFH and PC groups may suggest that fermentation effectively reduced infection and harmful bacteria. Similarly, *E. coli* was reduced in fermented feed groups which were more decreased in FFH group than the PC group. Patterson, Burkholder [54] recommended that studies in which there is no response to the growth promoting antibiotics should not be considered negative for the probiotic treatment. While the reduced *E. coli* in the FFH group is most likely due to the acidifying effects of fermented feed on the gut, fermented feeds create unfavorable environments for the proliferation of certain enteropathogens like *E. coli* [50], which is consistent with previous research that found adding fermented soyabean meal to chicken diets decreased harmful bacteria like *Escherichia coli* [51]. However very less differences between FFL and control groups suggests that the fermented feed preparation in chickens have been of minor magnitude because of the good condition of the birds. Similarly, *Lactobacillus* produces lactic acid, which is required for antimicrobial activity, as it inhibits the development and growth of Gram-negative bacteria’s virulence factors [55]. The combination of *P. acidilactici* and *L. fermentum* has the potential to eliminate *Anaerovibrio* by promoting the production of lactic acid, which filters through the gram-negative bacteria’s outer membrane [56]. *Lactobacillus aviarus* levels were significantly higher in the FFH and FFL fermented feed groups than in the PC and NC control groups (*P* < 0.05; Fig. 6). Due to the fact that *Lactobacillus* sp. is well-known for its beneficial effects on humans and animals, a high level of *Lactobacillus* sp. is associated with chicken health [57]. Additionally, previous research has demonstrated that feeding *Lactobacillus* strains significantly increases *Lactobacillus* diversity in the ileum and cecum of broilers [58, 59]. Beneficial bacteria were increased in FFL and FFH groups as a result of these changes in the cecal microbiota.

## Conclusion

These findings suggested that the FFA diet may modulate cecal microbiota by reducing pathogenic microorganisms such as phylum *Delsulfobacterota* and class *Desulfovibriona* and *Negativicutes* and improve beneficial microorganisms like *Lactobacillaceae* family, *Lactobacillus aviarus* genus and *Lactobacillus* spp. While FFA diet also affect immunity, and gene expression related to immunity. This study may provide an alternative method for improving broiler intestinal health. Although fermented feed did not have the same growth promoting effect as antibiotics in terms of feed efficiency, it was significantly better at reducing chicken mortality than antibiotics. Due to the scarcity of data, future studies must be consistent in their approach and cognizant of how feed fermentation ingredients affect the broiler population’s microbiota.

## Methods

The Animal Care and Use Committee of the South China Agricultural University reviewed and approved the protocol (Project number 2019B020218001). Experimental animals in Guangzhou, China, were treated humanely at all times and in accordance with the Ministry of Science and Technology’s Guide for Experimental Animals.

### Preparation of fermented feed

The *Lactobacillus casei* (C37M41) strain used in this study was screened in our laboratory. *Lactobacillus casei* (C37M41) was cultured for 18 hours at 37 °C with shaking at 200 rpm in de Man, Rogosa, and Sharp (MRS) liquid medium. The basal substrate was composed of corn, soybean meal, and wheat bran in proportions of 6:2:2. Additionally, 5% yeast extract and 5% molasses were added and poured into a 500 mL Erlenmeyer flask fitted with a glass stopper, supplemented with sterile water to achieve a moisture content of 40%. *Lactobacillus casei* (C37M41) (1 × 109 cfu/g) was added to the mixed substrate and fermented at 37 °C for 24 hours. The fermented mixture was placed in a one-way valve plastic bag (Rou Duoduo Biotechnology Co., Beijing, China) and inoculated with *Lactobacillus casei* (1 × 10^9^ cfu/g) a second time, and incubated at 37 °C in anaerobic conditions (the second-stage of fermentation). Controls were uninoculated flasks. All experimental procedures were identical for uninoculated samples except that sterile medium (LB and MRS) was added instead of bacteria. Triplicates of inoculated and uninoculated samples (control) were set up. The inoculated feed fermented for 14 days at 37 °C under anaerobic conditions in a dark anaerobic box. Moisture samples were collected at different inoculation times for microbial, pH, and lactic acid analysis.

Fermented feed was added to the basal diet, mixed evenly, and then made into pellets. The main content in the fermented feed was total bacteria count 2 × 10^9^ cfu/g, lactic acid 20 mg/g, acetic acid 5 mg/g, succinic acid 7 mg/g, and pyruvate 0.1 mg/g. Evaluation of the LAB count was performed according the ISO 15214:1998 method, described in detail by Bartkiene et al. [60]. The number of microorganisms was counted and expressed as log_10_ of colony-forming units per gram (CFU g^− 1^). All results are expressed as the mean of three determinations.

### Birds, diets, and experimental design

The test diet was formulated based on National Research Council (NRC) NRC [61] and NY/T 33–2004 and combined with the AA broiler feeding manual. Two hundred eighty-eight day-old Arbor Acre broilers, both male and female, were randomly assigned to one of four groups (Each group has 6 replicates, with each replicate containing 12 chickens). The groups were negative control (NC; basal diet), positive control (PC; basal diet +antibiotic virginiamycin 15 ppm) as this dosage for chicken’s were used and tested in other experiment [62], fermented feed additive low dose (FFL; basal diet + 0.3 kg/t *L. casei* fermented feed additive), and fermented feed additive high dose (FFH; 3 kg/t *L. casei* fermented feed additive). Broiler chicks were fed and watered ad libitum, with a 243 L:1D light regime maintained. The experiment lasted 42 days and was divided into two phases: a starter phase (0 to 21 days) and a finisher phase (22 to 42 days) (22 to 42 day). The basic diet’s composition and nutrient content are listed in Table 2. Pelletized feed was used in the experiment, and pellets were fed.

**Table 2:** Test diet formula and nutritional level

Items	Starter diet	Grower diet
Ingredient%	0 ~ 21 d	21 ~ 42 d
Corn	56.59	59.96
Soybean meal	25.95	20
Cotton meal	4.5	4.42
Wheat meal	4	5
Wheat middlings	2	2
Oil	2.49	4.5
Calcium hydrogen phosphate	1.82	1.58
Mountain flour	1.35	1.27
Salt	0.35	0.35
Lysine	0.35	0.35
Methionine	0.23	0.21
Threonine	0.05	0.04
Premix of trace elements^a^	0.2	0.2
Vitamin premix^b^	0.02	0.02
Choline chloride	0.1	0.1
Total	100	100
Nutrition level ^c^		
Metabolizable energy (kcal/kg)	2980	3160
Crude protein (%)	21.95	19.95
Calcium (%)	1	0.9
Available phosphorus (%)	0.45	0.4
Lysine (%)	1.30	1.15
Methionine (%)	0.58	0.54
Methionine+Cystine(%)	0.94	0.87
Threonine (%)	0.84	0.75
Tryptophan (%)	0.23	0.2

*Note*: ^a^ Premix of trace elements (provides milligrams per kilogram of feed): Cu 8, Zn 75, Fe 80, Mn 100, Se 0.15, I 0.35

^b^Vitamin premix (provided per kilogram of feed): Vitamin A 12500 IU, Vitamin D3 2500 IU; The following are mg: Vitamin E 18.75, Vitamin K3 2.65, Vitamin B12, Vitamin B2 6, Vitamin B12 0.025, Biotin 0.0325, Folic acid 1.25, pantothenic acid 12, niacin 50

^c^ The results are calculated values

### Growth performance

The chicks’ body weight (BW) and feed intake were measured on the 21st and 42nd days. The feed conversion ratio (FCR) was calculated using the average daily feed intake (ADFI), average daily gain (ADG), and feed conversion ratio (FCR). Throughout the experiment, mortality was recorded on a daily basis.

### Sampling procedure

Six chickens were chosen at random from each group on the 21st and 42nd days to obtain serum samples. The blood samples were centrifuged and kept at –20 °C for further testing. After a 12-hour feed withdrawal, the bird’s body weight was measured and sacrificed.

After bleeding and defeathering, all eviscerated weight was determined once entirely internal organs had been removed (head, neck, claws, trachea, esophagus, stomach, gizzard, gizzard contents, intestines, spleen, pancreas heart, liver, glandular, abdominal fat, full craw and cutin membrane, and gonads). The entire thigh and breast muscle tissue were removed from the animal carcasses 30 minutes postmortem. The abdominal fat, thigh muscle, and breast muscle (including the entire visceral region and abdominal cavity) were weighed, and the weight percentage of fully eviscerated yield was calculated as a percentage of body weight. The percentages of breast muscle, thigh muscle, abdominal fat, and liver were calculated as a percentage of the eviscerated carcass weight.

The thymus, bursa, and spleen were immediately removed, dried, and individually weighed (g) for each individual, and the ratio of thymus, bursa, and spleen weight: body weight (%) was calculated, where organ index = (organ weight in grams) / (live weight, in grams) × 100. The middle section of the jejunum and ileum tissues were placed in a 2 mL centrifuge tube and quickly frozen in liquid nitrogen before being transferred to an ultra-low temperature refrigerator set to − 80 °C for inflammatory factor expression analysis. To examine intestinal morphology, 2 cm segments of the duodenum, jejunum, and ileum were collected and fixed in a 10% buffered formalin solution (PH = 7.4). Cecal contents were placed in a 1.5 mL centrifuge tube, quickly frozen in liquid nitrogen, and then transferred to an ultra-low temperature refrigerator at − 80 °C for storage.

### Inflammatory factors in the intestine

#### RNA extraction

The RNA from frozen jejunum and ileum was extracted and purified according to the manufacturer’s instructions using the RNEasy Mini kit (QIAGEN). To summarise, 600 ml of RLT buffer was used to homogenise the tissues. Tissue Ruptor, a handheld rotor-stator homogenizer (Qiagen Inc.), was used to elute total RNA, which was then stored at 80 °C. A spectrophotometer was used to measure the amount of RNA (NanoDrop Products, Wilmington, DE). Each sample’s total RNA (300 ng) was extracted. The probe and primer set for the 28S rRNA and the cytokines Interleukin 1 beta, Interleukin 6, Interleukin 10, Interleukin 4, and IL-1β/β-Actin were designed using the Primer Express software programme (PE Applied Biosystems, Foster City, CA).

The expression of chemokine and cytokine mRNA was quantified using a well-defined technique. The full complement of cytokines and chemokines in the chicken genome has been recently characterised [63], and a broad spectrum of avian cytokines can be quantified using quantitative reverse transcriptase-polymerase chain reaction (qRT-PCR) assays [64–66].

Table 3 contains probes and primers for chemokines and cytokines. The qRT-PCR was performed using one-step RT-PCR master mix reagents and TaqMan fast universal PCR master mix. For amplification and detection of specific products, the Applied Biosystems 7500 Fast real-time PCR system was used with the following cycle profile: one cycle of 48 °C for 30 min and 95 °C for 20 s followed by 40 cycles of 95 °C for 3 s and 60 °C for 30 s. The increased fluorescence detected by the 7500 Fast sequence detection system during PCR amplification was caused by the 5′ nuclease activity of the rTth DNA polymerase hydrolyzing the target-specific probes. The housekeeping gene 28S rRNA was used for normalization. The correction factor for each sample was calculated by dividing the sample’s mean threshold cycle (CT) value for the 28S rRNA-specific product by the sample’s overall mean CT value for the 28S rRNA-specific product. The corrected cytokine mean was determined as follows: average of each replicate × cytokine slope/28S slope × 28S correction factor.

**Table 3:** Real-time quantitative RT-PCR probes and primers

Gene	Sequence type-Probe/primer sequence	Accession number
IL-1β	forward 5′--TTCATTACCGTCCCGTTG-3′	(NM_204524.1)
	reverse 5′--GCTTTTATTTCTCCAGTCACA-3′	
IL-6	forward 5′--AAATCCCTCCTCGCCAATCT-3 reverse 5′--CCCTCACGGTCTTCTCCATAAA-3	(NM_204628.1)
IL-10	forward 5′--CGCTGTCACCGCTTCTTCA-3 reverse 5′--TCCCGTTCTCATCCATCTTCTC-3	(NM_001004414.2)
IL-4	forward 5′--GTGCCCACGCTGTGCTTAC −3 reverse 5′--AGGAAACCTCTCCCTGGATGTC-3	(NM_001007079.1)
IFNγ	forward 5′--GCCCTTCCTGTAACCAGATG-3 reverse 5′--ACACGACAGCCAAGTCAACG-3	(NM_205149.1)
TLR4	forward 5′--TTTCCAAGCACCAGATAGCAACA-3 reverse 5′--TTCCAGCACAAGCCCTGAAATTA-3	(NM_001030693.1)

For the genomic DNA sequence

Interleukin 1 beta, Toll-like receptor 4, Interleukin 6, Interleukin 10, Interleukin 4, Interferon gamma

### Intestinal morphology

The morphometry analysis was carried out using the methods described previously by Hamid et al. [67] briefly 6 birds per group was taken for intestinal morphometric analyses formalin-fixed samples were used that had already been prepared by sectioning 5 μm thickness and staining with haematoxylin and eosin. In each sample, 15 intact, well-oriented crypt-villus units were counted in each type of tissue. An image processing and analysis system (version 6.0, Image-Pro) was used to determine villus height and crypt depth (CD).

### Cecal microbiota

#### DNA extraction

Microbial DNA was extracted from cecum samples using a QIAamp Fast DNA stool mini kit (Qiagen) according to the manufacturer’s instructions, as previously described [68]. The quality and the quantity of harvested DNA were measured using a NanoDrop 2000 UV–vis spectrophotometer (Thermo Scientific, Wilmington, USA); additionally, 1% agarose gel electrophoresis was used to ensure DNA quality. 338 F (5--ACTCCTACGGGAGGCAGCA-3) and 806 R (5--GGACTACHVGGGTWTCTAAT-3) were used to amplify the V3-V4 hypervariable regions of the bacterial 16S rRNA gene. A thermocycler PCR system (Gene Amp 9700, ABI, USA) was used to conduct PCR and the reactions were performed in triplicates: 20 μL mixture, containing 4 μL of 5°ø FastPfu Buffer, 2 μL of 2.5 mmol/L dNTPs, 0.8 μL of each primer (5 μmol/L), 0.4 μL of FastPfu Polymerase, and 10 ng of template DNA. The AxyPrep DNA Gel Extraction Kit (Axygen Biosciences, Union City, CA, USA) was used to purify the extracted PCR products from a 2% agarose gel, which were then quantified using QuantiFluor-ST (Promega, USA) according to the manufacturer’s instructions as previously described by Hamid et al. [67].

### Pyrosequencing and bacterial data processing

Purified amplicons were pooled and sequenced in equimolar concentrations on an Illumina MiSeq PE300 platform, according to Majorbio Bio-Pharm Technology Co. Ltd., Shanghai, China (Illumina, San Diego, USA). Bioinformatics analysis was performed on the sequencing data. Trimmomatic and FLASH software quality-filtered the established raw fastq sequences using the following criteria: (i) bases with a score of less than < 20 were excluded, and at any site with an average quality, the reads were truncatedscore < 20 across a 50-bp sliding window; (ii) Trimmomatic software was used to delete truncated reads that were less than 50 bp in length; (iii) reads of up to 2 mismatched nucleotides and more than 0 barcode mismatches were discarded; (iv) using the FLASH programme, the merged reads were removed; additionally, sequences that overlapped longer than 10 bp were assembled using their overlap sequences; (v) readings that have not been assembled have been excluded. After this, high-quality sequences acquired were paired with samples based on their barcodes, clustering these reads with up to 97% similarities in operational taxonomic units using U search (version 7.1). Then, using the Ribosomal Database Project classifier algorithm, the chimaera and singletons were eliminated and allocated to taxa. A BLAST search for taxonomic classification was performed using QIIME at the 70% confidence level in the SILVA database (version 1.8.0). Alpha diversity indices (i.e., Shannon, and Simpson) were calculated by QIIME from rarefied samples using for richness and diversity indices of the bacterial community.

### Statistical analysis

ANOVA was used to analyse data on growth performance, production performance, haematological parameters, carcass characteristics, and immunological parameters using SPSS software (IBM Corp. IBM SPSS Statistics for Windows, Version 23.0. Armonk, NY, USA). When a significant interaction was determined, Duncan’s multiple-range test was used for multiple comparisons. The data are presented as mean ± SEM. *P* ≤ 0.05 was considered to be statistically significant. Prior to performing variance analysis, the data on broiler mortality were transformed using arc sine (angular) transformation values to ensure that the data had a more normal distribution [69]. The OTU-abundance data was normalised using the sequence number of the sample with the fewest sequences (i.e., XJAW2.2). The alpha and beta diversity analyses were then carried out on the normalized data. Alpha diversity was applied to analyze metrics of species diversity the Shannon index and the Simpson index, both of these indices were calculated with QIIME (Version 1.7.0). Community diversity was identified using the Shannon and Simpson indexes. Beta diversity analysis was used to evaluate differences in species complexity among the samples. Beta diversities based on both weighted and unweighted Unifrac were calculated by QIIME software (Version 1.7.0).

Vegan packages in R were used to investigate the taxonomic composition at the phylum, family, class, genus, and species levels. To assess group differences, the Kruskal–Wallis H test was used; R was used to manage principal coordinate analysis (PCA) of the complete diversity of microbial communities at the species level using the Bray–Curtis distance [70].

## Supplementary Information

**Additional file 1.**

## Data Availability

All data generated or analysed during this study are included in this published article [and its supplementary information files]. And further if required any other information related with the data involving in the manuscript can be obtained from the corresponding author upon reasonable request.

## Abbreviations

NC: Negative control basal diet
PC: Positive control basal diet +antibiotic
FFL: Basal diet + Fermented feed low dose
FFH: Basal diet + Fermented feed high dose
FCR: Feed conversion ratio
ADG: Average daily gain
ADFI: Average daily feed intake
BW: Body weight
SSF: Solid state fermentation
IL-1β: Interleukin 1 beta
IL-6: Interleukin 6
IL-10: Interleukin 10
IL-4: Interleukin 4
IFNγ: Interferon gamma
TLR4: Toll-like receptor 4
qRT-PCR: Quantitative real-time PCR
VH: Villus height
CD: Crypt depth
AGPs: Antibiotic growth promoters
GIT: Gastrointestinal tract
FFA: Fermented feed additives
E.coli: *Escherichia coli*

## References

[1] Sugiharto S, Ranjitkar S (2019). Recent advances in fermented feeds towards improved broiler chicken performance, gastrointestinal tract microecology and immune responses: A review. Anim Nutr.

[2] Xu F, Zeng X, Ding X (2012). Effects of replacing soybean meal with fermented rapeseed meal on performance, serum biochemical variables and intestinal morphology of broilers. Asian Australas J Anim Sci.

[3] Canibe N, Jensen BB (2012). Fermented liquid feed—microbial and nutritional aspects and impact on enteric diseases in pigs. Anim Feed Sci Technol.

[4] Niba A, Beal J, Kudi A, Brooks P. Potential of bacterial fermentation as a biosafe method of improving feeds for pigs and poultry. Afr J Biotechnol. 2009;8(9):1758–67.

[5] Missotten J, Michiels J, Dierick N, Ovyn A, Akbarian A, De Smet S (2013). Effect of fermented moist feed on performance, gut bacteria and gut histo-morphology in broilers. Br Poult Sci.

[6] Sun H, Tang J, Fang C, Yao X, Wu Y, Wang X (2013). Molecular analysis of intestinal bacterial microbiota of broiler chickens fed diets containing fermented cottonseed meal. Poult Sci.

[7] Pj X, Lx H, Zhang C, Yl Z (2016). Nutrient assessment of olive leaf residues processed by solid-state fermentation as an innovative feedstuff additive. J Appl Microbiol.

[8] Yang Y, Iji P, Choct M (2009). Dietary modulation of gut microflora in broiler chickens: a review of the role of six kinds of alternatives to in-feed antibiotics. World's Poult Sci J.

[9] Sopková D, Hertelyová Z, Andrejčáková Z, Vlčková R, Gancarčíková S, Petrilla V (2017). The application of probiotics and flaxseed promotes metabolism of n-3 polyunsaturated fatty acids in pigs. J Appl Anim Res.

[10] Aktas B, De Wolfe TJ, Tandee K, Safdar N, Darien BJ, Steele JL (2015). The effect of lactobacillus casei 32G on the mouse cecum microbiota and innate immune response is dose and time dependent. PLoS One.

[11] Ivory K, Chambers S, Pin C, Prieto E, Arques J, Nicoletti C (2008). Oral delivery of lactobacillus casei Shirota modifies allergen-induced immune responses in allergic rhinitis. Clin Exp Allergy.

[12] Yeh RH, Hsieh CW, Chen KL (2018). Screening lactic acid bacteria to manufacture two-stage fermented feed and pelleting to investigate the feeding effect on broilers. Poult Sci.

[13] Gao M, Cieślak A, Kierończyk B, Huang H, Yanza YR, Zaworska-Zakrzewska A (2020). Effects of raw and fermented rapeseed cake on growth performance, methane production, and breast meat fatty acid composition in broiler chickens. Animals..

[14] Bhargav S, Panda BP, Ali M, Javed S (2008). Solid-state fermentation: an overview. Chem Biochem Eng Q.

[15] Güngör E, Altop A, Öztürk E, Erener G (2017). Nutritional changes of sour cherry (Prunus cerasus) kernel subjected to Aspergillus Niger solid-state fermentation. J Tekirdag Agric Fac.

[16] Altop A (2019). The effects of diets supplemented with fermented or non-fermented cherry kernels (Prunus avium L.) on growth performance, ileal histology, caecum microflora, and some meat quality parameters in broiler chickens. Poult Sci.

[17] Gungor E, Erener G (2020). Effect of dietary raw and fermented sour cherry kernel (Prunus cerasus L.) on growth performance, carcass traits, and meat quality in broiler chickens. Poult Sci.

[18] Marcinčák S, Klempová T, Bartkovský M, Marcinčáková D, Zdolec N, Popelka P, et al. Effect of fungal solid-state fermented product in broiler chicken nutrition on quality and safety of produced breast meat. Biomed Res Int. 2018;2018;1–8. 10.1155/2018/2609548 PMC6151372 30276201

[19] Zhang Z, Shi L, Pang W, Liu W, Li J, Wang H (2016). Dietary fiber intake regulates intestinal microflora and inhibits ovalbumin-induced allergic airway inflammation in a mouse model. PLoS One.

[20] Alshelmani M, Loh T, Foo H, Sazili A, Lau W (2016). Effect of feeding different levels of palm kernel cake fermented by Paenibacillus polymyxa ATCC 842 on nutrient digestibility, intestinal morphology, and gut microflora in broiler chickens. Anim Feed Sci Technol.

[21] Zhang J, Zhu J, Sun J, Li Y, Wang P, Jiang R (2016). Effect of fermented feed on intestinal morphology, immune status, carcass and growth performance of Emei Black chickens. FASEB J.

[22] Loh T, Law F, Foo H, Goh Y, Zulkifli I (2007). Effects of feeding a fermented product on egg production, faecal microflora and faecal pH in laying hens. J Anim Feed Sci.

[23] Aljubori A, Idrus Z, Soleimani AF, Abdullah N, Juan BL (2017). Response of broiler chickens to dietary inclusion of fermented canola meal under heat stress condition. Ital J Anim Sci.

[24] Li L, Li W, Liu S, Wang H (2020). Probiotic fermented feed improved the production, health and nutrient utilisation of yellow-feathered broilers reared in high altitude in Tibet. Br Poult Sci.

[25] Li C-l, Wang J, Zhang H-J, Wu S-G, Hui Q-R, Yang C-B (2019). Intestinal morphologic and microbiota responses to dietary bacillus spp. in a broiler chicken model. Front Physiol.

[26] Skrede G, Herstad O, Sahlstrøm S, Holck A, Slinde E, Skrede A (2003). Effects of lactic acid fermentation on wheat and barley carbohydrate composition and production performance in the chicken. Anim Feed Sci Technol.

[27] Chiang G, Lu W, Piao X, Hu J, Gong L, Thacker P (2009). Effects of feeding solid-state fermented rapeseed meal on performance, nutrient digestibility, intestinal ecology and intestinal morphology of broiler chickens. Asian Australas J Anim Sci.

[28] Skrede G, Sahlstrøm S, Skrede A, Holck A, Slinde E (2001). Lactic acid fermentation of wheat and barley whole meal flour modifies carbohydrate composition and increases digestibility in mink (Mustela vison). Anim Feed Sci Technol.

[29] Mohnl M (2011). Poultry production: how probiotics can play a role. Feed Int.

[30] Drażbo A, Ognik K, Zaworska A, Ferenc K, Jankowski J (2018). The effect of raw and fermented rapeseed cake on the metabolic parameters, immune status, and intestinal morphology of turkeys. Poult Sci.

[31] Shamoto K, Yamauchi K (2000). Recovery responses of chick intestinal villus morphology to different refeeding procedures. Poult Sci.

[32] Liu T, She R, Wang K, Bao H, Zhang Y, Luo D (2008). Effects of rabbit sacculus rotundus antimicrobial peptides on the intestinal mucosal immunity in chickens. Poult Sci.

[33] Hou Y, Wang L, Yi D, Ding B, Yang Z, Li J (2013). N-acetylcysteine reduces inflammation in the small intestine by regulating redox, EGF and TLR4 signaling. Amino Acids.

[34] Jazi V, Ashayerizadeh A, Toghyani M, Shabani A, Tellez G (2018). Fermented soybean meal exhibits probiotic properties when included in Japanese quail diet in replacement of soybean meal. Poult Sci.

[35] Bermudez-Brito M, Muñoz-Quezada S, Gomez-Llorente C, Matencio E, Bernal MJ, Romero F (2013). Cell-free culture supernatant of Bifidobacterium breve CNCM I-4035 decreases pro-inflammatory cytokines in human dendritic cells challenged with salmonella typhi through TLR activation. PLoS One.

[36] Kullberg MC, Jankovic D, Gorelick PL, Caspar P, Letterio JJ, Cheever AW (2002). Bacteria-triggered CD4+ T regulatory cells suppress helicobacter hepaticus–induced colitis. J Exp Med.

[37] Cong Y, Weaver CT, Lazenby A, Elson CO (2002). Bacterial-reactive T regulatory cells inhibit pathogenic immune responses to the enteric flora. J Immunol.

[38] Zhang Y, Shi C, Wang C, Lu Z, Wang F, Feng J (2018). Effect of soybean meal fermented with Bacillus subtilis BS12 on growth performance and small intestinal immune status of piglets. Food Agric Immunol.

[39] SA A-F, El-Sanhoury M, El-Mednay N, Abdel-Azeem F. (2008). Thyroid activity, some blood constituents, organs morphology and performance of broiler chicks fed supplemental organic acids. Int. J Poult Sci.

[40] Kabir SL, Rahman M, Rahman M, Rahman M, Ahmed S (2004). The dynamics of probiotics on growth performance and immune response in broilers. Int J Poult Sci.

[41] Makram A, Galal A, Fathi M, El-Attar A (2010). Carcass characteristics and immunocompetence parameters of four commercial broiler strain chickens under summer season of Egypt. Int J Poult Sci.

[42] Tang J, Sun H, Yao X, Wu Y, Wang X, Feng J (2012). Effects of replacement of soybean meal by fermented cottonseed meal on growth performance, serum biochemical parameters and immune function of yellow-feathered broilers. Asian Australas J Anim Sci.

[43] Choi Y, Lee S, Oh J (2014). Effects of dietary fermented seaweed and seaweed fusiforme on growth performance, carcass parameters and immunoglobulin concentration in broiler chicks. Asian Australas J Anim Sci.

[44] Cooper MD, Peterson RD, South MA, Good RA (1966). The functions of the thymus system and the bursa system in the chicken. J Exp Med.

[45] Fasina Y, Classen H, Garlich J, Black B, Ferket P, Uni Z (2006). Response of Turkey poults to soybean lectin levels typically encountered in commercial diets. 2. Effect on intestinal development and lymphoid organs. Poult Sci.

[46] Cheng J, Yuan Y, Zhao F, Chen J, Chen P, Li Y, et al. Thymic T-cell production is associated with changes in the gut microbiota in young chicks. Front Immunol. 2021;12(3632). 10.3389/fimmu.2021.700603 PMC8461177 34566959

[47] Kumar BV, Connors TJ, Farber DL (2018). Human T cell development, localization, and function throughout life. Immunity..

[48] Van der Waaij D (1986). The influence of the intestinal microflora on the relative thymus weight. Med Microbiol Immunol.

[49] Fan P, Li L, Rezaei A, Eslamfam S, Che D, Ma X (2015). Metabolites of dietary protein and peptides by intestinal microbes and their impacts on gut. Curr Protein Pept Sci.

[50] Fan P, Liu P, Song P, Chen X, Ma X (2017). Moderate dietary protein restriction alters the composition of gut microbiota and improves ileal barrier function in adult pig model. Sci Rep.

[51] Yitbarek A, Weese JS, Alkie TN, Parkinson J, Sharif S (2018). Influenza A virus subtype H9N2 infection disrupts the composition of intestinal microbiota of chickens. FEMS Microbiol Ecol.

[52] Oakley BB, Kogut MH (2016). Spatial and temporal changes in the broiler chicken cecal and fecal microbiomes and correlations of bacterial taxa with cytokine gene expression. Front Vet Sci.

[53] Sutcliffe IC (2010). A phylum level perspective on bacterial cell envelope architecture. Trends Microbiol.

[54] Patterson J, Burkholder K (2003). Application of prebiotics and probiotics in poultry production. Poult Sci.

[55] Zhang Z, Lv J, Pan L, Zhang Y (2018). Roles and applications of probiotic lactobacillus strains. Appl Microbiol Biotechnol.

[56] Alakomi H-L, Skyttä E, Saarela M, Mattila-Sandholm T, Latva-Kala K, Helander I (2000). Lactic acid permeabilizes gram-negative bacteria by disrupting the outer membrane. Appl Environ Microbiol.

[57] Coeuret V, Gueguen M, Vernoux JP (2004). Numbers and strains of lactobacilli in some probiotic products. Int J Food Microbiol.

[58] Nakphaichit M, Thanomwongwattana S, Phraephaisarn C, Sakamoto N, Keawsompong S, Nakayama J (2011). The effect of including lactobacillus reuteri KUB-AC5 during post-hatch feeding on the growth and ileum microbiota of broiler chickens. Poult Sci.

[59] Chim-anage P, Hirunvong V, Sirirote P, Malaphan W, Yongsmith B, Isariyodom S (2008). Effect of feed supplementation of lactic acid bacteria on microbial changes in broiler intestine. Agric Nat Resour.

[60] Bartkiene E, Bartkevics V, Krungleviciute V, Pugajeva I, Zadeike D, Juodeikiene G (2017). Lactic acid bacteria combinations for wheat sourdough preparation and their influence on wheat bread quality and acrylamide formation. J Food Sci.

[61] NRC U (1994). Nutrient requirements of poultry.

[62] Samuel K, Zhang H, Wang J, Wu S, Yue H, Sun L (2015). Effects of dietary pyrroloquinoline quinone disodium on growth performance, carcass yield and antioxidant status of broiler chicks. Animal..

[63] Kaiser P (2007). The avian immune genome–a glass half-full or half-empty?. Cytogenet Genome Res.

[64] Smith CK, Kaiser P, Rothwell L, Humphrey T, Barrow PA, Jones MA (2005). Campylobacter jejuni-induced cytokine responses in avian cells. Infect Immun.

[65] Hong YH, Lillehoj HS, Lee SH, Dalloul RA, Lillehoj EP (2006). Analysis of chicken cytokine and chemokine gene expression following Eimeria acervulina and Eimeria tenella infections. Vet Immunol Immunopathol.

[66] Talpur MZ, Peng W, Zeng Y, Xie P, Li J, Zhang H, Shu G, Jiang Q. Effects of phenylpyruvate on the growth performance and intestinal microbiota in broiler chicken. Brit Poult Sci. 2022;1–10. 10.1080/00071668.2022.2061330. 10.1080/00071668.2022.2061330 35382668

[67] Hamid H, Zhang J, Li W, Liu C, Li M, Zhao L (2019). Interactions between the cecal microbiota and non-alcoholic steatohepatitis using laying hens as the model. Poult Sci.

[68] Dewar ML, Arnould JP, Dann P, Trathan P, Groscolas R, Smith S (2013). Interspecific variations in the gastrointestinal microbiota in penguins. Microbiologyopen..

[69] Zuidhof M, Robinson F, Feddes J, Hardin R, Wilson J, McKay R (1995). The effects of nutrient dilution on the well-being and performance of female broiler breeders. Poult Sci.

[70] Xia J, Wishart DS (2011). Web-based inference of biological patterns, functions and pathways from metabolomic data using MetaboAnalyst. Nat Protoc.

