# Supplementary material for: Influence of fermented feed additive on gut morphology, immune status, and microbiota in broilers
Source: BMC Vet Res. 2022 Jun 10;18:218. doi: 10.1186/s12917-022-03322-4 (PMC9185985; doi:10.1186/s12917-022-03322-4)
Supplement: Supplementary file 1 — Additional file 1. [file 12917_2022_3322_MOESM1_ESM.zip › test of IL-10.pdf]

"Table Analyzed" IL-10

"Column C"	FFH
vs.	vs.
"Column A"	NC

"Unpaired t test"
"	P value"	0.0202
"	P value summary"	*
"	Significantly different (P < 0.05)?" Yes
"	One- or two-tailed P value?" Two-tailed
"	t, df"	"t=2.758, df=10"

"How big is the difference?"
"	Mean of column A"	1.000
"	Mean of column C"	2.068
"	Difference between means (C - A) ± SEM"	"1.068 ± 0.3874"
"	95% confidence interval"	"0.2051 to 1.931"
"	R squared (eta squared)"	0.4319

"F test to compare variances"
"	F, DFn, Dfd"	"1.342, 5, 5"
"	P value"	0.7545
"	P value summary"	ns
"	Significantly different (P < 0.05)?" No

"Data analyzed"
"	Sample size, column A"	6
"	Sample size, column C"	6
