# Supplementary material for: Influence of fermented feed additive on gut morphology, immune status, and microbiota in broilers
Source: BMC Vet Res. 2022 Jun 10;18:218. doi: 10.1186/s12917-022-03322-4 (PMC9185985; doi:10.1186/s12917-022-03322-4)
Supplement: Supplementary file 1 — Additional file 1. [file 12917_2022_3322_MOESM1_ESM.zip › test of IL-10-1.pdf]

"Table Analyzed" IL-10

"Column D" FFL
vs. vs.
"Column A" NC

"Unpaired t test"
" P value" 0.1958
" P value summary" ns
" Significantly different (P < 0.05)?" No
" One- or two-tailed P value?" Two-tailed
" t, df" "t=1.397, df=9"

"How big is the difference?"
" Mean of column A" 1.000
" Mean of column D" 1.839
" Difference between means (D - A) ± SEM" "0.8390 ± 0.6005"
" 95% confidence interval" "-0.5194 to 2.197"
" R squared (eta squared)" 0.1783

"F test to compare variances"
" F, DFn, Dfd" "4.506, 4, 5"
" P value" 0.1300
" P value summary" ns
" Significantly different (P < 0.05)?" No

"Data analyzed"
" Sample size, column A" 6
" Sample size, column D" 5
