# Supplementary material for: Influence of fermented feed additive on gut morphology, immune status, and microbiota in broilers
Source: BMC Vet Res. 2022 Jun 10;18:218. doi: 10.1186/s12917-022-03322-4 (PMC9185985; doi:10.1186/s12917-022-03322-4)
Supplement: Supplementary file 1 — Additional file 1. [file 12917_2022_3322_MOESM1_ESM.zip › test of Rate of Liver.pdf]

```
"Table Analyzed" "Rate of Liver"

"Column D"       FFH
vs.     vs.
"Column A"       NC

"Unpaired t test"
"    P value"    0.4322
"    P value summary"    ns
"    Significantly different (P < 0.05)?" No
"    One- or two-tailed P value?" Two-tailed
"    t, df"      "t=0.7988, df=24"

"How big is the difference?"
"    Mean of column A"   2.711
"    Mean of column D"   2.851
"    Difference between means (D - A) ± SEM"       "0.1397 ± 0.1749"
"    95% confidence interval"     "-0.2212 to 0.5006"
"    R squared (eta squared)"     0.02590

"F test to compare variances"
"    F, DFn, Dfd"        "2.688, 11, 13"
"    P value"    0.0934
"    P value summary"    ns
"    Significantly different (P < 0.05)?" No

"Data analyzed"
"    Sample size, column A"       14
"    Sample size, column D"       12
```
