# Supplementary material for: Influence of fermented feed additive on gut morphology, immune status, and microbiota in broilers
Source: BMC Vet Res. 2022 Jun 10;18:218. doi: 10.1186/s12917-022-03322-4 (PMC9185985; doi:10.1186/s12917-022-03322-4)
Supplement: Supplementary file 1 — Additional file 1. [file 12917_2022_3322_MOESM1_ESM.zip › test of Spleen.pdf]

"Table Analyzed" Spleen

"Column D"   FFH
vs.   vs.
"Column A"   NC

"Unpaired t test"
"   P value"   0.4058
"   P value summary"   ns
"   Significantly different (P < 0.05)?" No
"   One- or two-tailed P value?" Two-tailed
"   t, df"   "t=0.8461, df=24"

"How big is the difference?"
"   Mean of column A"   0.8810
"   Mean of column D"   0.8108
"   Difference between means (D - A) ± SEM"   "-0.07025 ± 0.08303"
"   95% confidence interval"   "-0.2416 to 0.1011"
"   R squared (eta squared)"   0.02897

"F test to compare variances"
"   F, DFn, Dfd"   "1.190, 11, 13"
"   P value"   0.7564
"   P value summary"   ns
"   Significantly different (P < 0.05)?" No

"Data analyzed"
"   Sample size, column A"   14
"   Sample size, column D"   12
