# Supplementary material for: Influence of fermented feed additive on gut morphology, immune status, and microbiota in broilers
Source: BMC Vet Res. 2022 Jun 10;18:218. doi: 10.1186/s12917-022-03322-4 (PMC9185985; doi:10.1186/s12917-022-03322-4)
Supplement: Supplementary file 1 — Additional file 1. [file 12917_2022_3322_MOESM1_ESM.zip › test of Thymus Index-3.pdf]

"Table Analyzed" "Thymus Index"

"Column D"	FFH
vs.	vs.
"Column C"	FFL

"Unpaired t test"
"	P value"	0.9212
"	P value summary"	ns
"	Significantly different (P < 0.05)?"	No
"	One- or two-tailed P value?"	Two-tailed
"	t, df"	"t=0.1000, df=22"

"How big is the difference?"
"	Mean of column C"	1.148
"	Mean of column D"	1.163
"	Difference between means (D - C) ± SEM"	"0.01442 ± 0.1441"
"	95% confidence interval"	"-0.2844 to 0.3133"
"	R squared (eta squared)"	0.0004547

"F test to compare variances"
"	F, DFn, Dfd"	"4.648, 11, 11"
"	P value"	0.0171
"	P value summary"	*
"	Significantly different (P < 0.05)?"	Yes

"Data analyzed"
"	Sample size, column C"	12
"	Sample size, column D"	12
