# Supplementary material for: Influence of fermented feed additive on gut morphology, immune status, and microbiota in broilers
Source: BMC Vet Res. 2022 Jun 10;18:218. doi: 10.1186/s12917-022-03322-4 (PMC9185985; doi:10.1186/s12917-022-03322-4)
Supplement: Supplementary file 1 — Additional file 1. [file 12917_2022_3322_MOESM1_ESM.zip › test of Thymus Index-4.pdf]

"Table Analyzed" "Thymus Index"

"Column C" FFL
vs. vs.
"Column B" PC

"Unpaired t test"
" P value" 0.1175
" P value summary" ns
" Significantly different (P < 0.05)?" No
" One- or two-tailed P value?" Two-tailed
" t, df" "t=1.624, df=24"

"How big is the difference?"
" Mean of column B" 1.338
" Mean of column C" 1.148
" Difference between means (C - B) ± SEM" "-0.1895 ± 0.1167"
" 95% confidence interval" "-0.4304 to 0.05139"
" R squared (eta squared)" 0.09897

"F test to compare variances"
" F, DFn, Dfd" "2.838, 13, 11"
" P value" 0.0915
" P value summary" ns
" Significantly different (P < 0.05)?" No

"Data analyzed"
" Sample size, column B" 14
" Sample size, column C" 12
