# Supplementary material for: Influence of fermented feed additive on gut morphology, immune status, and microbiota in broilers
Source: BMC Vet Res. 2022 Jun 10;18:218. doi: 10.1186/s12917-022-03322-4 (PMC9185985; doi:10.1186/s12917-022-03322-4)
Supplement: Supplementary file 1 — Additional file 1. [file 12917_2022_3322_MOESM1_ESM.zip › test of TLR-1.pdf]

"Table Analyzed" TLR4

"Column B" PC
vs. vs.
"Column A" NC

"Unpaired t test"
" P value" 0.0152
" P value summary" *
" Significantly different (P < 0.05)?" Yes
" One- or two-tailed P value?" Two-tailed
" t, df" "t=2.829, df=12"

"How big is the difference?"
" Mean of column A" 1.000
" Mean of column B" 1.982
" Difference between means (B - A) ± SEM" "0.9821 ± 0.3472"
" 95% confidence interval" "0.2257 to 1.738"
" R squared (eta squared)" 0.4001

"F test to compare variances"
" F, DFn, Dfd" "4.284, 6, 6"
" P value" 0.1000
" P value summary" ns
" Significantly different (P < 0.05)?" No

"Data analyzed"
" Sample size, column A" 7
" Sample size, column B" 7
