# Supplementary material for: Influence of fermented feed additive on gut morphology, immune status, and microbiota in broilers
Source: BMC Vet Res. 2022 Jun 10;18:218. doi: 10.1186/s12917-022-03322-4 (PMC9185985; doi:10.1186/s12917-022-03322-4)
Supplement: Supplementary file 1 — Additional file 1. [file 12917_2022_3322_MOESM1_ESM.zip › test of TLR-4.pdf]

"Table Analyzed" TLR4

"Column D" FFH
vs. vs.
"Column A" NC

"Unpaired t test"
" P value" 0.0618
" P value summary" ns
" Significantly different (P < 0.05)?" No
" One- or two-tailed P value?" Two-tailed
" t, df" "t=2.103, df=10"

"How big is the difference?"
" Mean of column A" 1.000
" Mean of column D" 1.642
" Difference between means (D - A) ± SEM" "0.6423 ± 0.3054"
" 95% confidence interval" "-0.03819 to 1.323"
" R squared (eta squared)" 0.3067

"F test to compare variances"
" F, DFn, Dfd" "2.760, 4, 6"
" P value" 0.2570
" P value summary" ns
" Significantly different (P < 0.05)?" No

"Data analyzed"
" Sample size, column A" 7
" Sample size, column D" 5
