# Supplementary material for: Influence of fermented feed additive on gut morphology, immune status, and microbiota in broilers
Source: BMC Vet Res. 2022 Jun 10;18:218. doi: 10.1186/s12917-022-03322-4 (PMC9185985; doi:10.1186/s12917-022-03322-4)
Supplement: Supplementary file 1 — Additional file 1. [file 12917_2022_3322_MOESM1_ESM.zip › (species).pdf]

NC NC NC NC PC PC PC
PC FFL FFL FFL FFL FFH
FFH FFH FFH FFH FFH FFH
Bacteroides_plebeius 0.023156650 0.173457001
0.067166734 0.018487970 0.234056460 0.711755735
0.160758194 0.200628715 0.201064459 0.017056242
0.347754365 0.519001525 0.020511065
0.278035420 0.040461888 0.611192381 0.032960876
0.048492017 0.268480189 0.560117028
0.326589685 0.319617791 0.040928756
Lactobacillus_aviarius 0.017180740 0.009243985
0.004295185 0.006691774 0.001276106 0.017429736
0.008714868 0.001618476 0.006536151 0.003454823
0.000933736 0.002023094 0.023872514
0.010022098 0.015966884 0.001213857 0.006473902
0.001556226 0.034174733 0.005415668
0.019328333 0.002832332 0.044165707
Bacteroides_sp_Marseille-P3166 0.208596595 0.265741231
0.003517072 0.011142581 0.259049457 0.000871487
0.001369479 0.006131532 0.460113916 0.038687790
0.004170687 0.000746989 0.137352548
0.201686949 0.235488188 0.010800212 0.181829500
0.460113916 0.004419683 0.008216876
0.016153631 0.088891656 0.002863457
Bacteroides_barnesiae 0.000031100 0.001431728
0.001618476 0.000093400 0.000248996 0.000155623
0.000248996 0.000062200 0.000031100 0.001649600
0.000186747 0.000186747 0.000000000
0.000902611 0.214136761 0.000000000 0.353481279
0.000000000 0.000000000 0.000653615
0.000684740 0.000000000 0.000000000
Bacteroides_salanitronis 0.000000000 0.000684740
0.000746989 0.000995985 0.000000000 0.000000000
0.000000000 0.000000000 0.000000000 0.000560242
0.000000000 0.000000000 0.000000000
0.000000000 0.001182732 0.000000000 0.000622491
0.000871487 0.000000000 0.000000000
0.000000000 0.000000000 0.000404619
Helicobacter_pullorum 0.001462853 0.001493977
0.016776121 0.001276106 0.017772106 0.000684740
0.000622491 0.001027109 0.002272091 0.010364468
0.001618476 0.000342370 0.079305301
0.002396589 0.001587351 0.004326310 0.017367487
0.014815276 0.044943820 0.000809238
0.023499020 0.002956830 0.000684740
Parasutterella_secunda 0.005882536 0.001338355
0.011329329 0.019857450 0.010426717 0.002334340
0.002956830 0.000591366 0.001462853 0.016278129
0.003423698 0.000684740 0.037505058
0.004699804 0.008652619 0.005011049 0.007656634
0.006442778 0.004762053 0.002614460
0.000466868 0.009741978 0.001338355
Bacteroides_uniformis 0.000715864 0.004762053
0.003641570 0.006131532 0.005477917 0.003921691

0.004450808    0.000622491    0.001774098    0.009057238
0.004606430    0.001369479        0.002676710
0.000871487    0.003019079    0.001276106    0.009243985
0.001244981        0.000248996    0.001742974
0.001929721    0.003236951    0.017056242
Lactobacillus_oris    0.000062200    0.000155623
0.000840362    0.000155623    0.002116468    0.000124498
0.000186747    0.000217872    0.000715864    0.004917676
0.002521087    0.000404619        0.003952815
0.000217872    0.002583336    0.000155623    0.000715864
0.000466868        0.000248996    0.001431728
0.000809238    0.000186747    0.004513057
Desulfovibrio_piger    0.039839397    0.000933736
0.000995985    0.000404619    0.000280121    0.000124498
0.000373494    0.000560242    0.001649600    0.000902611
0.000373494    0.000248996        0.001369479
0.000964860    0.016776121    0.002489962    0.029599427
0.000155623        0.000715864    0.000155623
0.000497992    0.001244981    0.000217872
