# Supplementary material for: Influence of fermented feed additive on gut morphology, immune status, and microbiota in broilers
Source: BMC Vet Res. 2022 Jun 10;18:218. doi: 10.1186/s12917-022-03322-4 (PMC9185985; doi:10.1186/s12917-022-03322-4)
Supplement: Supplementary file 1 — Additional file 1. [file 12917_2022_3322_MOESM1_ESM.zip › (order).pdf]

NC NC NC NC NC NC NC
PC PC PC PC FFL FFL FFL FFL
FFH FFH FFH FFH
Lactobacillales 0.018581344 0.014192785 0.018207850
0.014099412 0.069563323 0.019515080 0.018394597
0.009586355 0.013290174 0.017678733 0.019266084
0.012294189 0.078527187 0.015593389
0.094898690 0.004544181 0.014846400 0.014255034
0.038189797 0.018985963 0.041613496
0.046468922 0.130256155
Bacteroidales 0.365962215 0.586697376 0.344673037
0.397740359 0.583367052 0.749883283 0.384138940
0.602290765 0.759096144 0.356500358 0.401195182
0.678608111 0.375953189 0.521927231
0.556848953 0.667403281 0.624980547 0.585452395
0.352205173 0.726228641 0.591366056
0.458900059 0.264402876
Veillonellales-Selenomonadales 0.143110585 0.002209842
0.094867565 0.032369510 0.021818295 0.007563261
0.003423698 0.048958884 0.018519095 0.022534159
0.003548196 0.014410657 0.004979925
0.107317377 0.015904634 0.006193781 0.010146597
0.101279218 0.003019079 0.003205827
0.024339382 0.001213857 0.001151608
Lachnospirales 0.078496063 0.147219023 0.124217996
0.161038314 0.061533194 0.037754054 0.124964985
0.126801332 0.075476983 0.142456970 0.331507361
0.061159700 0.140091506 0.156214012
0.120887672 0.099193875 0.082915746 0.128326434
0.155249152 0.080207912 0.132590495
0.294655918 0.252824551
Oscillospirales 0.241152853 0.058420741 0.094680818
0.108406735 0.126365589 0.111550313 0.157272246
0.095396682 0.049052258 0.259858695 0.125680849
0.035357465 0.109745090 0.082791248
0.056210900 0.109682841 0.062000062 0.033489993
0.220641788 0.060381587 0.071337421
0.062809300 0.080674780
Acidaminococcales 0.026922718 0.042951850
0.050639609 0.051044228 0.052258085 0.020728936
0.099940863 0.027638582 0.023934763 0.031778144
0.051075352 0.122599521 0.026144605
0.013725917 0.007563261 0.024028137 0.042329360
0.013757042 0.004419683 0.023218899
0.014753027 0.006878521 0.002023094
Campylobacterales 0.001711849 0.001680725
0.017523110 0.002645585 0.017896604 0.001369479
0.000622491 0.006069283 0.002334340 0.010675714
0.001774098 0.000653615 0.079336425
0.006598400 0.012387563 0.004544181 0.017554234
0.019857450 0.045597435 0.001027109
0.024277133 0.003019079 0.000902611
Clostridia_UCG-014 0.020946808 0.015749012
0.076504093 0.045846432 0.021009057 0.007220891

0.023748016	0.015344393	0.007750008	0.032525133
0.011453827	0.012387563		0.028727940
0.013072302	0.015002023	0.015935759	0.034766099
0.008403623		0.037785178	0.018892589
0.003050204	0.017429736	0.072831398
Clostridia_vadinBB60_group	0.007999004	0.024214884
0.043947835	0.050421737	0.010862461	0.016029133
0.073018146	0.014317283	0.010520091	0.022191789
0.004513057	0.005384544		0.021538174
0.007999004	0.025055246	0.010208846	0.024059261
0.009368483		0.012605434	0.027296212
0.016247004	0.018394597	0.025646612
Burkholderiales	0.007034144	0.013103427	0.013850416
0.022596408	0.012294189	0.003454823	0.005602415
0.001836347	0.002178717	0.018923714	0.005477917
0.002552211		0.044010084	0.005415668
0.009804227	0.005975910	0.012294189	0.009928725
	0.040181767	0.003828317	0.006691774
0.013227925	0.015344393
