# Supplementary material for: Influence of fermented feed additive on gut morphology, immune status, and microbiota in broilers
Source: BMC Vet Res. 2022 Jun 10;18:218. doi: 10.1186/s12917-022-03322-4 (PMC9185985; doi:10.1186/s12917-022-03322-4)
Supplement: Supplementary file 1 — Additional file 1. [file 12917_2022_3322_MOESM1_ESM.zip › (genus).pdf]

NC NC Nc NC
PC PC PC PC FFL
FFL FFL FFL FFH FFH FFH FFH
Lactobacillus 0.017989978 0.013103427 0.017242989
0.012636559 0.062529179 0.019421706 0.017896604
0.009461857 0.013072302 0.016029133 0.017523110
0.009586355 0.078402689 0.014815276
0.094431822 0.004450808 0.013788166 0.010395593
0.037940801 0.018861465 0.041488997
0.045877556 0.121572411
Bacteroides 0.237417909 0.456098852 0.082293255
0.048243020 0.521989480 0.722742694 0.183230104
0.209779327 0.688101092 0.107690871 0.363814622
0.523732454 0.167854586 0.498895079
0.500544679 0.630676336 0.586852999 0.524323820
0.277412929 0.576083912 0.353823648
0.419838775 0.064801270
Alistipes 0.091692863 0.104453920 0.184288338
0.285069563 0.052351458 0.019110461 0.114227022
0.379501385 0.041986990 0.231753245 0.029568303
0.147810389 0.168414828 0.011142581
0.031560273 0.028821314 0.015157646 0.051822341
0.055993028 0.127766193 0.024246008
0.031933767 0.154502163
Megamonas 0.143110585 0.001960845 0.094649693
0.032276137 0.021818295 0.007563261 0.003392574
0.048958884 0.018519095 0.022222914 0.003454823
0.014317283 0.004979925 0.107099505
0.015468891 0.006193781 0.009928725 0.100905724
0.003019079 0.003205827 0.024339382
0.001213857 0.001058234
Barnesiella 0.030688786 0.016682748 0.065268138
0.041053254 0.005602415 0.005695789 0.054748047
0.008528121 0.026393601 0.005758038 0.005664664
0.004668679 0.012294189 0.008901615
0.009959849 0.002707834 0.012854431 0.004762053
0.002272091 0.012263065 0.198232127
0.002552211 0.018954838
Faecalibacterium 0.189330511 0.010893585 0.029163684
0.033707865 0.087055308 0.081608516 0.071804289
0.059603473 0.023343397 0.181673877 0.089016154
0.005042174 0.028354446 0.036135578
0.012076317 0.062155685 0.029537178 0.008341374
0.085436833 0.021444801 0.016402627
0.016527125 0.002645585
torques_group 0.014690778 0.074761119 0.051230975
0.040524137 0.018736967 0.009710853 0.066077376
0.035606461 0.025366491 0.026020106 0.049270130
0.026051231 0.032182763 0.074107504
0.045099443 0.032992001 0.023125525 0.052787202
0.050234990 0.025304242 0.045255066
0.133088487 0.109371596
CHKCI001 0.020884559 0.023467895 0.004201811
0.028821314 0.001369479 0.001649600 0.014441782

0.004793178 0.003610445 0.036166703 0.130038283
0.002676710 0.008185751 0.016807246
0.003205827 0.002085343 0.012947804 0.021507050
0.006380528 0.001493977 0.005384544
0.010644589 0.001929721
Phascolarctobacterium 0.026922718 0.042951850
0.050639609 0.051044228 0.052258085 0.020728936
0.099940863 0.027638582 0.023934763 0.031778144
0.051075352 0.122599521 0.026144605
0.013725917 0.007532136 0.024028137 0.042329360
0.013757042 0.004419683 0.023218899
0.014753027 0.006878521 0.002023094
Helicobacter 0.001525102 0.001525102 0.016776121
0.001276106 0.017772106 0.000684740 0.000622491
0.001027109 0.002334340 0.010426717 0.001618476
0.000342370 0.079305301 0.002396589
0.001587351 0.004326310 0.017367487 0.014815276
0.044943820 0.000809238 0.023499020
0.002956830 0.000809238
