# Supplementary material for: Influence of fermented feed additive on gut morphology, immune status, and microbiota in broilers
Source: BMC Vet Res. 2022 Jun 10;18:218. doi: 10.1186/s12917-022-03322-4 (PMC9185985; doi:10.1186/s12917-022-03322-4)
Supplement: Supplementary file 1 — Additional file 1. [file 12917_2022_3322_MOESM1_ESM.zip › (family).pdf]

NC NC NC NC
PC PC PC PC FFL FFL FFL
FFL FFH FFH FFH FFH
Lactobacillaceae 0.017989978 0.013103427 0.017242989
0.012636559 0.062529179 0.019421706 0.017896604
0.009461857 0.013072302 0.016029133 0.017523110
0.009586355 0.078402689 0.014815276
0.094431822 0.004450808 0.013788166 0.010395593
0.037940801 0.018861465 0.041488997
0.045877556 0.121572411
Bacteroidaceae 0.237417909 0.456098852 0.082293255
0.048243020 0.521989480 0.722742694 0.183230104
0.209779327 0.688101092 0.107690871 0.363814622
0.523732454 0.167854586 0.498895079
0.500544679 0.630676336 0.586852999 0.524323820
0.277412929 0.576083912 0.353823648
0.419838775 0.064801270
Rikenellaceae 0.092377603 0.105294282 0.184910828
0.289022379 0.052569330 0.019328333 0.120949921
0.380123876 0.042049239 0.232220113 0.029692801
0.148152759 0.173923869 0.011329329
0.032182763 0.028976937 0.015188770 0.051977964
0.066793240 0.129291294 0.027514084
0.031996016 0.157925861
Selenomonadaceae 0.143110585 0.001960845 0.094680818
0.032276137 0.021818295 0.007563261 0.003392574
0.048958884 0.018519095 0.022222914 0.003517072
0.014317283 0.004979925 0.107099505
0.015468891 0.006193781 0.009928725 0.101092471
0.003019079 0.003205827 0.024339382
0.001213857 0.001058234
Lachnospiraceae 0.078153693 0.147032276 0.124031249
0.160820443 0.061533194 0.037691805 0.124964985
0.126458962 0.075227987 0.142176850 0.331507361
0.061128575 0.140060382 0.156182888
0.120825423 0.098913754 0.082728999 0.128233060
0.155031280 0.079647670 0.132434872
0.293971179 0.252700053
Ruminococcaceae 0.209499206 0.023965888 0.054467926
0.055712907 0.093933829 0.087459927 0.112141679
0.072924772 0.033334371 0.203461048 0.104360547
0.023218899 0.053129571 0.047776152
0.019950823 0.074356500 0.037473933 0.014939774
0.100656728 0.026455850 0.034548227
0.032743005 0.038376545
Barnesiellaceae 0.030875533 0.016931744 0.067011111
0.041084379 0.005664664 0.005695789 0.054779171
0.008808242 0.026393601 0.005820287 0.005664664
0.004668679 0.012356438 0.008932740
0.009959849 0.002707834 0.013010053 0.004793178
0.002427713 0.012294189 0.198294376
0.002583336 0.019110461
Acidaminococcaceae 0.026922718 0.042951850
0.050639609 0.051044228 0.052258085 0.020728936

0.099940863 0.027638582 0.023934763 0.031778144
0.051075352 0.122599521 0.026144605
0.013725917 0.007563261 0.024028137 0.042329360
0.013757042 0.004419683 0.023218899
0.014753027 0.006878521 0.002023094
Oscillospiraceae 0.023654642 0.024775125 0.025957857
0.044819322 0.025646612 0.019577329 0.029568303
0.014784151 0.009928725 0.040181767 0.011920695
0.008092378 0.047807277 0.030097420
0.027109465 0.029723925 0.017865480 0.014348408
0.110990071 0.027233963 0.031342401
0.022471910 0.030066295
Helicobacteraceae 0.001525102 0.001525102
0.016776121 0.001276106 0.017772106 0.000684740
0.000622491 0.001027109 0.002334340 0.010426717
0.001618476 0.000342370 0.079305301
0.002396589 0.001587351 0.004326310 0.017367487
0.014815276 0.044943820 0.000809238
0.023499020 0.002956830 0.000809238
