# Supplementary material for: Influence of fermented feed additive on gut morphology, immune status, and microbiota in broilers
Source: BMC Vet Res. 2022 Jun 10;18:218. doi: 10.1186/s12917-022-03322-4 (PMC9185985; doi:10.1186/s12917-022-03322-4)
Supplement: Supplementary file 1 — Additional file 1. [file 12917_2022_3322_MOESM1_ESM.zip › 2-test of TLR-2.pdf]

"Table Analyzed" TLR4

"Column B"   PC
vs.   vs.
"Column A"   NC

"Unpaired t test"
"   P value"   0.0152
"   P value summary"   *
"   Significantly different (P < 0.05)?" Yes
"   One- or two-tailed P value?" Two-tailed
"   t, df"   "t=2.829, df=12"

"How big is the difference?"
"   Mean of column A"   1.000
"   Mean of column B"   1.982
"   Difference between means (B - A) ± SEM"   "0.9821 ± 0.3472"
"   95% confidence interval"   "0.2257 to 1.738"
"   R squared (eta squared)"   0.4001

"F test to compare variances"
"   F, DFn, Dfd"   "4.284, 6, 6"
"   P value"   0.1000
"   P value summary"   ns
"   Significantly different (P < 0.05)?" No

"Data analyzed"
"   Sample size, column A"   7
"   Sample size, column B"   7
