# Supplementary material for: Influence of fermented feed additive on gut morphology, immune status, and microbiota in broilers
Source: BMC Vet Res. 2022 Jun 10;18:218. doi: 10.1186/s12917-022-03322-4 (PMC9185985; doi:10.1186/s12917-022-03322-4)
Supplement: Supplementary file 1 — Additional file 1. [file 12917_2022_3322_MOESM1_ESM.zip › (class).pdf]

NC NC NC NC NC NC NC
PC PC PC PC PC PC PC FFL
FFL FFL FFL FFL FFH FFH FFH FFH
Bacilli 0.020946808 0.017056242 0.022876529
0.019795201 0.070652681 0.020791186 0.020884559
0.013196800 0.014846400 0.022689782 0.022720906
0.020635563 0.083133618 0.019483955
0.097046282 0.006380528 0.017274114 0.017025117
0.046188801 0.021475925 0.042609481
0.049425752 0.151794329
Bacteroidia 0.366709204 0.588689346 0.359830683
0.408447197 0.585825889 0.750817019 0.399514457
0.603846992 0.760310000 0.361822652 0.402564661
0.679572971 0.382053596 0.522705344
0.559992530 0.670702481 0.626879143 0.587444365
0.357278471 0.727286875 0.596096984
0.463070746 0.269227178
Clostridia 0.358990320 0.261632793 0.353419030
0.380217249 0.223567494 0.179775281 0.392853808
0.260232189 0.150704971 0.470571758 0.482772573
0.125960970 0.319119798 0.265678982
0.232593607 0.247471132 0.212269289 0.188925892
0.460207289 0.198512248 0.231846618
0.417130941 0.461732391
Negativicutes 0.170095552 0.045161692 0.145538299
0.083413738 0.074076380 0.028292197 0.103364562
0.076753089 0.042547231 0.054312304 0.054623549
0.137134676 0.031124529 0.121043294
0.023467895 0.030221918 0.052475956 0.115036260
0.007438762 0.026424725 0.039092409
0.008092378 0.003174702
Campylobacteria 0.001711849 0.001680725 0.017523110
0.002645585 0.017896604 0.001369479 0.000622491
0.006069283 0.002334340 0.010675714 0.001774098
0.000653615 0.079336425 0.006598400
0.012387563 0.004544181 0.017554234 0.019857450
0.045597435 0.001027109 0.024277133
0.003019079 0.000902611
Gammaproteobacteria 0.007314264 0.013134551
0.013850416 0.032245012 0.012356438 0.003517072
0.011578325 0.002023094 0.002209842 0.019701827
0.006691774 0.003143577 0.044010084
0.005509042 0.009804227 0.006318279 0.012543185
0.011733948 0.040212892 0.003890566
0.006691774 0.013321299 0.016589374
Desulfovibrionia 0.046064303 0.002178717 0.005353419
0.004637555 0.001213857 0.000560242 0.002085343
0.001493977 0.002832332 0.001742974 0.001058234
0.001867472 0.005322295 0.002987955
0.019515080 0.009212861 0.031840393 0.011235955
0.002987955 0.001151608 0.002552211
0.002272091 0.002925706
Synergistia 0.003517072 0.000529117 0.002552211
0.000280121 0.000000000 0.000093400 0.001898596

0.000000000 0.004450808 0.000155623 0.000093400
0.000093400 0.000062200 0.003268076
0.005073298 0.000093400 0.001493977 0.006038159
0.000093400 0.000031100 0.000000000
0.003081328 0.000871487
Vampirivibrionia 0.001680725 0.000560242 0.005789162
0.005166672 0.001058234 0.000186747 0.003392574
0.001929721 0.000186747 0.001774098 0.000155623
0.000280121 0.011951819 0.000497992
0.002427713 0.000995985 0.002552211 0.002552211
0.002676710 0.000622491 0.009866476
0.000466868 0.003859442
Verrucomicrobiae 0.000404619 0.000031100 0.000062200
0.000000000 0.000000000 0.000000000 0.000000000
0.000000000 0.000000000 0.000124498 0.000000000
0.000000000 0.000186747 0.013912665
0.000000000 0.000000000 0.000062200 0.000093400
0.000093400 0.000342370 0.000000000
0.000000000 0.000093400
