# Supplementary material for: Influence of fermented feed additive on gut morphology, immune status, and microbiota in broilers
Source: BMC Vet Res. 2022 Jun 10;18:218. doi: 10.1186/s12917-022-03322-4 (PMC9185985; doi:10.1186/s12917-022-03322-4)
Supplement: Supplementary file 1 — Additional file 1. [file 12917_2022_3322_MOESM1_ESM.zip › test of IL-1(beta).pdf]

"Table Analyzed" IL-1β

"Column B" PC
vs. vs.
"Column A" NC

"Unpaired t test"
" P value" 0.1485
" P value summary" n
" Significantly different (P < 0.05)?" sNo
" One- or two-tailed P value?" Two-tailed
" t, df" "t=1.544, df=12"

"How big is the difference?"
" Mean of column A" 1.0001
" Mean of column B" .6410
" Difference between means (B - A) ± SEM" ".6411 ± 0.4151-"
" 95% confidence interval" "0.2634 to 1.5460"
" R squared (eta squared)" .16581

"F test to compare variances"
" F, DFn, Dfd" ".136, 6, 60"
" P value" .8812n
" P value summary" sN
" Significantly different (P < 0.05)?" o7

"Data analyzed"
" Sample size, column A" 7
" Sample size, column B" I
