# Supplementary material for: Influence of fermented feed additive on gut morphology, immune status, and microbiota in broilers
Source: BMC Vet Res. 2022 Jun 10;18:218. doi: 10.1186/s12917-022-03322-4 (PMC9185985; doi:10.1186/s12917-022-03322-4)
Supplement: Supplementary file 1 — Additional file 1. [file 12917_2022_3322_MOESM1_ESM.zip › test of Bursa Index-2.pdf]

"Table Analyzed" "Bursa Index"

"Column C" FFL
vs. vs.
"Column B" PC

"Unpaired t test"
" P value" 0.5302
" P value summary" ns
" Significantly different (P < 0.05)?" No
" One- or two-tailed P value?" Two-tailed
" t, df" "t=0.6369, df=24"

"How big is the difference?"
" Mean of column B" 0.4037
" Mean of column C" 0.3874
" Difference between means (C - B) ± SEM" "-0.01630 ± 0.02559"
" 95% confidence interval" "-0.06911 to 0.03652"
" R squared (eta squared)" 0.01662

"F test to compare variances"
" F, DFn, Dfd" "1.267, 11, 13"
" P value" 0.6764
" P value summary" ns
" Significantly different (P < 0.05)?" No

"Data analyzed"
" Sample size, column B" 14
" Sample size, column C" 12
