# Supplementary material for: Influence of fermented feed additive on gut morphology, immune status, and microbiota in broilers
Source: BMC Vet Res. 2022 Jun 10;18:218. doi: 10.1186/s12917-022-03322-4 (PMC9185985; doi:10.1186/s12917-022-03322-4)
Supplement: Supplementary file 1 — Additional file 1. [file 12917_2022_3322_MOESM1_ESM.zip › test of Bursa Index-1.pdf]

```
"Table Analyzed" "Bursa Index"

"Column D"       FFH
vs.     vs.
"Column C"       FFL

"Unpaired t test"
"    P value"    0.5485
"    P value summary"    ns
"    Significantly different (P < 0.05)?" No
"    One- or two-tailed P value?" Two-tailed
"    t, df"      "t=0.6094, df=22"

"How big is the difference?"
"    Mean of column C"   0.3874
"    Mean of column D"   0.4036
"    Difference between means (D - C) ± SEM"       "0.01617 ±
0.02653"
"    95% confidence interval"     "-0.03885 to 0.07118"
"    R squared (eta squared)"     0.01660

"F test to compare variances"
"    F, DFn, Dfd"        "1.303, 11, 11"
"    P value"    0.6688
"    P value summary"    ns
"    Significantly different (P < 0.05)?" No

"Data analyzed"
"    Sample size, column C"       12
"    Sample size, column D"       12
```
