# Supplementary material for: Influence of fermented feed additive on gut morphology, immune status, and microbiota in broilers
Source: BMC Vet Res. 2022 Jun 10;18:218. doi: 10.1186/s12917-022-03322-4 (PMC9185985; doi:10.1186/s12917-022-03322-4)
Supplement: Supplementary file 1 — Additional file 1. [file 12917_2022_3322_MOESM1_ESM.zip › test of Bursa Index.pdf]

"Table Analyzed" "Bursa Index"

"Column D"	FFH
vs.	vs.
"Column A"	NC

"Unpaired t test"
"	P value"	0.6303
"	P value summary"	ns
"	Significantly different ($P < 0.05$)?" No
"	One- or two-tailed P value?" Two-tailed
"	t, df"	"t=0.4875, df=24"

"How big is the difference?"
"	Mean of column A"	0.3899
"	Mean of column D"	0.4036
"	Difference between means (D - A) ± SEM"	"0.01373 ± 0.02815"
"	95% confidence interval"	"-0.04438 to 0.07183"
"	R squared (eta squared)"	0.009807

"F test to compare variances"
"	F, DFn, Dfd"	"1.732, 13, 11"
"	P value"	0.3682
"	P value summary"	ns
"	Significantly different ($P < 0.05$)?" No

"Data analyzed"
"	Sample size, column A"	14
"	Sample size, column D"	12
