# Supplementary material for: Influence of fermented feed additive on gut morphology, immune status, and microbiota in broilers
Source: BMC Vet Res. 2022 Jun 10;18:218. doi: 10.1186/s12917-022-03322-4 (PMC9185985; doi:10.1186/s12917-022-03322-4)
Supplement: Supplementary file 1 — Additional file 1. [file 12917_2022_3322_MOESM1_ESM.zip › test of Breast Muscle-2.pdf]

```
"Table Analyzed" "Breast Muscle"

"Column C"       FFL
vs.     vs.
"Column B"       PC

"Unpaired t test"
"    P value"    0.1482
"    P value summary"    ns
"    Significantly different (P < 0.05)?" No
"    One- or two-tailed P value?" Two-tailed
"    t, df"      "t=1.494, df=24"

"How big is the difference?"
"    Mean of column B"   26.33
"    Mean of column C"   25.29
"    Difference between means (C - B) ± SEM"       "-1.040 ± 0.6964"
"    95% confidence interval"     "-2.478 to 0.3968"
"    R squared (eta squared)"     0.08509

"F test to compare variances"
"    F, DFn, Dfd"        "1.084, 11, 13"
"    P value"    0.8787
"    P value summary"    ns
"    Significantly different (P < 0.05)?" No

"Data analyzed"
"    Sample size, column B"       14
"    Sample size, column C"       12
```
