# Supplementary material for: Influence of fermented feed additive on gut morphology, immune status, and microbiota in broilers
Source: BMC Vet Res. 2022 Jun 10;18:218. doi: 10.1186/s12917-022-03322-4 (PMC9185985; doi:10.1186/s12917-022-03322-4)
Supplement: Supplementary file 1 — Additional file 1. [file 12917_2022_3322_MOESM1_ESM.zip › test of Breast Muscle-1.pdf]

"Table Analyzed" "Breast Muscle"

"Column D" FFH
vs. vs.
"Column B" PC

"Unpaired t test"
" P value" 0.1077
" P value summary" ns
" Significantly different (P < 0.05)?" No
" One- or two-tailed P value?" Two-tailed
" t, df" "t=1.671, df=24"

"How big is the difference?"
" Mean of column B" 26.33
" Mean of column D" 25.09
" Difference between means (D - B) ± SEM" "-1.236 ± 0.7398"
" 95% confidence interval" "-2.763 to 0.2907"
" R squared (eta squared)" 0.1042

"F test to compare variances"
" F, DFn, Dfd" "1.375, 11, 13"
" P value" 0.5780
" P value summary" ns
" Significantly different (P < 0.05)?" No

"Data analyzed"
" Sample size, column B" 14
" Sample size, column D" 12
