# Supplementary material for: Influence of fermented feed additive on gut morphology, immune status, and microbiota in broilers
Source: BMC Vet Res. 2022 Jun 10;18:218. doi: 10.1186/s12917-022-03322-4 (PMC9185985; doi:10.1186/s12917-022-03322-4)
Supplement: Supplementary file 1 — Additional file 1. [file 12917_2022_3322_MOESM1_ESM.zip › test of Breast Muscle.pdf]

"Table Analyzed" "Breast Muscle"

"Column D" FFH
vs. vs.
"Column A" NC

"Unpaired t test"
" P value" 0.2035
" P value summary" ns
" Significantly different (P < 0.05)?" No
" One- or two-tailed P value?" Two-tailed
" t, df" "t=1.307, df=24"

"How big is the difference?"
" Mean of column A" 26.39
" Mean of column D" 25.09
" Difference between means (D - A) ± SEM" "-1.297 ± 0.9922"
" 95% confidence interval" "-3.345 to 0.7507"
" R squared (eta squared)" 0.06647

"F test to compare variances"
" F, DFn, Dfd" "1.983, 13, 11"
" P value" 0.2626
" P value summary" ns
" Significantly different (P < 0.05)?" No

"Data analyzed"
" Sample size, column A" 14
" Sample size, column D" 12
