# Supplementary material for: Influence of fermented feed additive on gut morphology, immune status, and microbiota in broilers
Source: BMC Vet Res. 2022 Jun 10;18:218. doi: 10.1186/s12917-022-03322-4 (PMC9185985; doi:10.1186/s12917-022-03322-4)
Supplement: Supplementary file 1 — Additional file 1. [file 12917_2022_3322_MOESM1_ESM.zip › test of All Eviscerate-3.pdf]

"Table Analyzed" "All Eviscerate"

"Column C"	FFL
vs.	vs.
"Column B"	PC

"Unpaired t test"
"	P value"	0.4528
"	P value summary"	ns
"	Significantly different (P < 0.05)?" No
"	One- or two-tailed P value?" Two-tailed
"	t, df"	"t=0.7632, df=24"

"How big is the difference?"
"	Mean of column B"	73.17
"	Mean of column C"	72.19
"	Difference between means (C - B) ± SEM"	"-0.9824 ± 1.287"
"	95% confidence interval"	"-3.639 to 1.674"
"	R squared (eta squared)"	0.02369

"F test to compare variances"
"	F, DFn, Dfd"	"2.436, 11, 13"
"	P value"	0.1296
"	P value summary"	ns
"	Significantly different (P < 0.05)?" No

"Data analyzed"
"	Sample size, column B"	14
"	Sample size, column C"	12
