# Supplementary material for: Influence of fermented feed additive on gut morphology, immune status, and microbiota in broilers
Source: BMC Vet Res. 2022 Jun 10;18:218. doi: 10.1186/s12917-022-03322-4 (PMC9185985; doi:10.1186/s12917-022-03322-4)
Supplement: Supplementary file 1 — Additional file 1. [file 12917_2022_3322_MOESM1_ESM.zip › test of Spleen-1.pdf]

"Table Analyzed" Spleen

"Column C" FFL
vs. vs.
"Column A" NC

"Unpaired t test"
" P value" 0.4448
" P value summary" ns
" Significantly different (P < 0.05)?" No
" One- or two-tailed P value?" Two-tailed
" t, df" "t=0.7769, df=24"

"How big is the difference?"
" Mean of column A" 0.8810
" Mean of column C" 0.9666
" Difference between means (C - A) ± SEM" "0.08558 ± 0.1102"
" 95% confidence interval" "-0.1418 to 0.3129"
" R squared (eta squared)" 0.02453

"F test to compare variances"
" F, DFn, Dfd" "2.993, 11, 13"
" P value" 0.0639
" P value summary" ns
" Significantly different (P < 0.05)?" No

"Data analyzed"
" Sample size, column A" 14
" Sample size, column C" 12
