# Supplementary material for: Influence of fermented feed additive on gut morphology, immune status, and microbiota in broilers
Source: BMC Vet Res. 2022 Jun 10;18:218. doi: 10.1186/s12917-022-03322-4 (PMC9185985; doi:10.1186/s12917-022-03322-4)
Supplement: Supplementary file 1 — Additional file 1. [file 12917_2022_3322_MOESM1_ESM.zip › test of IL-6.pdf]

```
"Table Analyzed" IL-6

"Column B"       PC
vs.     vs.
"Column A"       NC

"Unpaired t test"
"    P value"    0.1310
"    P value summary"    ns
"    Significantly different (P < 0.05)?" No
"    One- or two-tailed P value?" Two-tailed
"    t, df"      "t=1.662, df=9"

"How big is the difference?"
"    Mean of column A"   0.4518
"    Mean of column B"   0.9585
"    Difference between means (B - A) ± SEM"       "0.5067 ± 0.3050"
"    95% confidence interval"     "-0.1832 to 1.197"
"    R squared (eta squared)"     0.2347

"F test to compare variances"
"    F, DFn, Dfd"        "3.509, 4, 5"
"    P value"    0.2011
"    P value summary"    ns
"    Significantly different (P < 0.05)?" No

"Data analyzed"
"    Sample size, column A"       6
"    Sample size, column B"       5
```
