# Supplementary material for: Influence of fermented feed additive on gut morphology, immune status, and microbiota in broilers
Source: BMC Vet Res. 2022 Jun 10;18:218. doi: 10.1186/s12917-022-03322-4 (PMC9185985; doi:10.1186/s12917-022-03322-4)
Supplement: Supplementary file 1 — Additional file 1. [file 12917_2022_3322_MOESM1_ESM.zip › test of IFN.pdf]

"Table Analyzed" IFN-γ

"Column D" PC
vs. vs.
"Column A" NC

"Unpaired t test"
" P value" 0.2593
" P value summary" ns
" Significantly different (P < 0.05)?" No
" One- or two-tailed P value?" Two-tailed
" t, df" "t=1.190, df=11"

"How big is the difference?"
" Mean of column A" 1.000
" Mean of column D" 1.363
" Difference between means (D - A) ± SEM" "0.3634 ± 0.3055"
" 95% confidence interval" "-0.3090 to 1.036"
" R squared (eta squared)" 0.1140

"F test to compare variances"
" F, DFn, Dfd" "1.965, 5, 6"
" P value" 0.4347
" P value summary" ns
" Significantly different (P < 0.05)?" No

"Data analyzed"
" Sample size, column A" 7
" Sample size, column D" 6
