# Supplementary material for: Influence of fermented feed additive on gut morphology, immune status, and microbiota in broilers
Source: BMC Vet Res. 2022 Jun 10;18:218. doi: 10.1186/s12917-022-03322-4 (PMC9185985; doi:10.1186/s12917-022-03322-4)
Supplement: Supplementary file 1 — Additional file 1. [file 12917_2022_3322_MOESM1_ESM.zip › test of Breast Muscle-4.pdf]

```
"Table Analyzed" "Breast Muscle"

"Column B"       PC
vs.     vs.
"Column A"       NC

"Unpaired t test"
"    P value"    0.9464
"    P value summary"    ns
"    Significantly different (P < 0.05)?" No
"    One- or two-tailed P value?" Two-tailed
"    t, df"      "t=0.06789, df=26"

"How big is the difference?"
"    Mean of column A"   26.39
"    Mean of column B"   26.33
"    Difference between means (B - A) ± SEM"       "-0.06086 ±
0.8964"
"    95% confidence interval"     "-1.903 to 1.782"
"    R squared (eta squared)"     0.0001772

"F test to compare variances"
"    F, DFn, Dfd"        "2.728, 13, 13"
"    P value"    0.0818
"    P value summary"    ns
"    Significantly different (P < 0.05)?" No

"Data analyzed"
"    Sample size, column A"       14
"    Sample size, column B"       14
```
