# Supplementary material for: Influence of fermented feed additive on gut morphology, immune status, and microbiota in broilers
Source: BMC Vet Res. 2022 Jun 10;18:218. doi: 10.1186/s12917-022-03322-4 (PMC9185985; doi:10.1186/s12917-022-03322-4)
Supplement: Supplementary file 1 — Additional file 1. [file 12917_2022_3322_MOESM1_ESM.zip › TLR4.pdf]

NC	PC	FFL	FFH
0.376665294	1.482807595	0.314243496	0.795193031
1.165245670	2.708233013	1.671221076	1.232325749
0.659744481	3.378709419	0.641281821	1.869125537
0.889655407	1.147738563	1.854084129	2.541339870
1.417409368	2.271970958	1.216704397	1.773477089
1.489707474	1.357931504	15.974073240*	13.848255510*
1.001572305	1.527227383		
