# Supplementary material for: Influence of fermented feed additive on gut morphology, immune status, and microbiota in broilers
Source: BMC Vet Res. 2022 Jun 10;18:218. doi: 10.1186/s12917-022-03322-4 (PMC9185985; doi:10.1186/s12917-022-03322-4)
Supplement: Supplementary file 1 — Additional file 1. [file 12917_2022_3322_MOESM1_ESM.zip › Thigh Muscle.pdf]

NC	PC	FFL	FFH
34.463	32.337	29.206	32.426
32.297	30.540	32.419	30.781
36.880	32.311	31.774	31.505
35.847	32.349	34.580	33.595
31.427	30.248	31.500	33.113
32.205	31.791	33.741	33.921
30.371	31.244		
		38.852	38.061
31.657	31.130	36.155	36.334
31.791	33.071	29.812	27.891
34.764	32.687	28.330	27.837
35.164	35.334	30.637	36.350
32.290	27.396	32.897	32.668
33.541	32.303		
32.842	33.777		
