# Supplementary material for: Influence of fermented feed additive on gut morphology, immune status, and microbiota in broilers
Source: BMC Vet Res. 2022 Jun 10;18:218. doi: 10.1186/s12917-022-03322-4 (PMC9185985; doi:10.1186/s12917-022-03322-4)
Supplement: Supplementary file 1 — Additional file 1. [file 12917_2022_3322_MOESM1_ESM.zip › Rate of Liver-1.pdf]

2.858	1.920	3.336	4.089
2.159	2.442	2.764	2.731
3.217	2.408	2.509	2.586
3.031	3.287	2.606	2.891
2.908	2.571	2.202	2.646
2.565	3.128	2.897	2.829
2.741	1.975		
		2.037	3.811
3.173	2.192	2.390	2.716
2.362	2.115	2.687	2.403
2.653	2.317	2.382	2.408
2.488	2.276	2.340	2.791
3.028	2.494	3.068	2.310
2.431	3.151		
2.343	2.275		
