# Supplementary material for: Influence of fermented feed additive on gut morphology, immune status, and microbiota in broilers
Source: BMC Vet Res. 2022 Jun 10;18:218. doi: 10.1186/s12917-022-03322-4 (PMC9185985; doi:10.1186/s12917-022-03322-4)
Supplement: Supplementary file 1 — Additional file 1. [file 12917_2022_3322_MOESM1_ESM.zip › IL-6.pdf]

NC	PC	FFL	FFH
0.225505474	0.100911601	0.832585311	1.890792451
0.195621672	20.631725800*	10.405691900*	0.103956345
0.201100867	27.857307120*	0.377304554	0.357641541
1.014361984	1.764773123	1.599931244	1.114709381
4.289164014*	1.418633791	0.589747228	1.290340657
0.747897523	0.788264415	94.398232340*	103.533949900*
0.326348467	0.719937552		
