# Supplementary material for: Influence of fermented feed additive on gut morphology, immune status, and microbiota in broilers
Source: BMC Vet Res. 2022 Jun 10;18:218. doi: 10.1186/s12917-022-03322-4 (PMC9185985; doi:10.1186/s12917-022-03322-4)
Supplement: Supplementary file 1 — Additional file 1. [file 12917_2022_3322_MOESM1_ESM.zip › IL-4.pdf]

NC	PC.	FFL	FFH
1.048041406	0.242852284	4.039085394	3.741631678
0.153508215	16.692948090*	17.135984320*	0.063892604
0.162724004	24.809687740*	0.198037709	0.118286929
1.869124829	1.839814826	1.393525920	1.523975472
2.437532526	1.422734179	0.211456173	0.799129057
1.043504449	0.786192333	51.688403770*	221.291258100*
0.285564571	0.371593822		
