# Supplementary material for: Influence of fermented feed additive on gut morphology, immune status, and microbiota in broilers
Source: BMC Vet Res. 2022 Jun 10;18:218. doi: 10.1186/s12917-022-03322-4 (PMC9185985; doi:10.1186/s12917-022-03322-4)
Supplement: Supplementary file 1 — Additional file 1. [file 12917_2022_3322_MOESM1_ESM.zip › IL-1(beta).pdf]

NC	PC	FFL	FFH
0.727589455	0.499851191	0.608240826	0.787996519
0.438546926	0.807946142	0.377775185	0.839999010
0.408429283	2.443016455	1.183122292	0.459982774
2.448772353*	1.625846060	1.745106253	2.038084358
1.597860495	1.674441203	0.435997198	1.753312439
0.675504331	1.697844128	4.839647799*	4.392580148*
0.703297157	2.738753719		
