# Supplementary material for: Influence of fermented feed additive on gut morphology, immune status, and microbiota in broilers
Source: BMC Vet Res. 2022 Jun 10;18:218. doi: 10.1186/s12917-022-03322-4 (PMC9185985; doi:10.1186/s12917-022-03322-4)
Supplement: Supplementary file 1 — Additional file 1. [file 12917_2022_3322_MOESM1_ESM.zip › 2-IL-10.pdf]

NC	PC	FFL	FFH
0.598643503	0.537345340	2.727432867	7.028620432*
0.403787484	0.788890811	3.106109168	0.312996687
0.815287602	0.237181324	1.474211728	1.116774253
1.592893179	1.442599524	1.246996306	3.141867545
10.656301060*	1.926263594	1.903229078	3.294980084
1.945106226	1.129738397	1.951260716	1.328428853
0.644282005	3.058000136		
