# Supplementary material for: Influence of fermented feed additive on gut morphology, immune status, and microbiota in broilers
Source: BMC Vet Res. 2022 Jun 10;18:218. doi: 10.1186/s12917-022-03322-4 (PMC9185985; doi:10.1186/s12917-022-03322-4)
Supplement: Supplementary file 1 — Additional file 1. [file 12917_2022_3322_MOESM1_ESM.zip › IFN.pdf]

NC	PC	FFL	FFH
0.431679416	1.533745789	0.611153921	0.900593020
1.596545966	0.978455412	0.874088134	2.558049250
1.264732723	0.745524647	0.463057363	1.316040302
0.385009631	1.188700701	1.274677795	1.026841426
1.272459453	0.848883320	1.914687132	0.841722645
0.859165755	1.405958427	1.367506128	1.537123637
1.190407056	0.864899987		
