# Supplementary material for: Influence of fermented feed additive on gut morphology, immune status, and microbiota in broilers
Source: BMC Vet Res. 2022 Jun 10;18:218. doi: 10.1186/s12917-022-03322-4 (PMC9185985; doi:10.1186/s12917-022-03322-4)
Supplement: Supplementary file 1 — Additional file 1. [file 12917_2022_3322_MOESM1_ESM.zip › Breast Muscle.pdf]

NC	PC	FFL	FFH
23.518	28.335	25.857	25.651
28.435	27.565	25.581	27.053
27.343	26.068	28.168	27.966
30.929	24.154	26.587	26.415
25.572	27.349	23.742	24.125
25.331	24.416	23.898	21.725
27.548	24.886		
		27.492	23.213
23.774	26.288	26.677	27.169
25.640	27.456	22.068	24.084
29.215	28.759	25.596	22.296
20.169	27.909	24.221	26.653
24.444	25.700	23.592	24.780
29.348	22.899		
28.211	26.841		
