# Supplementary material for: Influence of fermented feed additive on gut morphology, immune status, and microbiota in broilers
Source: BMC Vet Res. 2022 Jun 10;18:218. doi: 10.1186/s12917-022-03322-4 (PMC9185985; doi:10.1186/s12917-022-03322-4)
Supplement: Supplementary file 1 — Additional file 1. [file 12917_2022_3322_MOESM1_ESM.zip › All Eviscerate.pdf]

NC	PC	FFL	FFH
73.095	76.740	68.081	67.646
74.209	73.989	75.306	70.666
65.369	71.300	72.254	72.055
67.008	75.125	64.925	73.385
74.077	72.190	74.111	70.420
71.090	71.904	70.804	74.724
73.295	72.315		
		79.682	64.975
68.787	72.083	67.408	63.470
78.516	72.664	74.591	73.385
68.239	72.779	72.819	75.452
68.277	66.749	73.867	69.321
76.963	75.991	72.423	71.809
61.285	74.991		
69.016	75.583		
