# Supplementary material for: Influence of fermented feed additive on gut morphology, immune status, and microbiota in broilers
Source: BMC Vet Res. 2022 Jun 10;18:218. doi: 10.1186/s12917-022-03322-4 (PMC9185985; doi:10.1186/s12917-022-03322-4)
Supplement: Supplementary file 1 — Additional file 1. [file 12917_2022_3322_MOESM1_ESM.zip › phylum.pdf]

NC NC NC PC
PC PC PC FFL FFL FFL FFL
FFH FFH FFH FFH
Firmicutes 0.550343926 0.324410968 0.522892091
0.484702294 0.368483302 0.229201033 0.527747518
0.350337701 0.209001214 0.548165209 0.560241526
0.283855707 0.434124934 0.406455227
0.353668026 0.284820567 0.282112733 0.321485263
0.514052725 0.246910890 0.313828628
0.475053690 0.617292788
Bacteroidota 0.366709204 0.588689346 0.359830683
0.408447197 0.585825889 0.750817019 0.399514457
0.603846992 0.760310000* 0.361822652 0.402564661
0.679572971 0.382053596 0.522705344
0.559992530 0.670702481 0.626879143 0.587444365
0.357278471 0.727286875 0.596096984
0.463070746 0.269258302
Campilobacterota 0.001711849 0.001680725 0.017523110
0.002645585 0.017896604 0.001369479 0.000622491
0.006069283 0.002334340 0.010675714 0.001774098
0.000653615 0.079336425 0.006598400
0.012387563 0.004544181 0.017554234 0.019857450
0.045597435 0.001027109 0.024277133
0.003019079 0.000902611
Proteobacteria 0.007314264 0.013134551 0.013850416
0.032587382 0.012356438 0.003517072 0.011733948
0.002023094 0.002209842 0.020106446 0.006691774
0.003143577 0.044010084 0.005509042
0.009804227 0.006318279 0.012543185 0.011733948
0.040212892 0.003921691 0.006691774
0.013321299 0.018083351
Desulfobacterota 0.046064303 0.002178717 0.005353419
0.004637555 0.001213857 0.000560242 0.002085343
0.001493977 0.002832332 0.001742974 0.001058234
0.001867472 0.005322295 0.002987955
0.019515080 0.009212861 0.031840393 0.011235955
0.002987955 0.001151608 0.002552211
0.002272091 0.002925706
Synergistota 0.003517072 0.000529117 0.002552211
0.000280121 0.000000000 0.000093400 0.001898596
0.000000000 0.004450808 0.000155623 0.000093400
0.000093400 0.000062200 0.003268076
0.005073298 0.000093400 0.001493977 0.006038159
0.000093400 0.000031100 0.000000000
0.003081328 0.000871487
unidentified_Bacteria 0.005695789 0.007065268
0.022067291 0.014006038 0.004513057 0.003423698
0.014659653 0.004481932 0.003268076 0.011733948
0.004201811 0.003081328 0.009461857
0.004855427 0.008372498 0.004015064 0.009461857
0.004357434 0.009399608 0.007501012
0.008061253 0.007469887 0.010769087
Cyanobacteria 0.001680725 0.000560242 0.005789162
0.005166672 0.001058234 0.000186747 0.003392574

0.001929721	0.000186747	0.001774098	0.000155623
0.000280121		0.011951819	0.000497992
0.002427713	0.000995985	0.002552211	0.002552211
	0.002676710	0.000622491	0.009866476
0.000466868	0.003859442
Verrucomicrobiota		0.000435743	0.000031100
0.000062200	0.000000000	0.000062200	0.000000000
0.000000000	0.000000000	0.000000000	0.000124498
0.000000000	0.000000000		0.000248996
0.013943789	0.000000000	0.000000000	0.000062200
0.000093400		0.000093400	0.000342370
0.000031100	0.000062200	0.000124498
Actinobacteriota	0.000560242	0.000715864	0.000435743
0.001027109	0.000124498	0.000248996	0.000684740
0.000342370	0.000871487	0.001960845	0.001836347
0.005509042		0.000684740	0.001058234
0.000342370	0.000560242	0.000280121	0.002645585
	0.000778113	0.000684740	0.000404619
0.002178717	0.005695789
