# Supplementary material for: Influence of fermented feed additive on gut morphology, immune status, and microbiota in broilers
Source: BMC Vet Res. 2022 Jun 10;18:218. doi: 10.1186/s12917-022-03322-4 (PMC9185985; doi:10.1186/s12917-022-03322-4)
Supplement: Supplementary file 1 — Additional file 1. [file 12917_2022_3322_MOESM1_ESM.zip › test of CD.pdf]

"Table Analyzed" (CD)

"Column B" FFH
vs. vs.
"Column A" NC

"Unpaired t test"
" P value" 0.9698
" P value summary" ns
" Significantly different (P < 0.05)?" No
" One- or two-tailed P value?" Two-tailed
" t, df" "t=0.03865, df=12"

"How big is the difference?"
" Mean of column A" 108.8
" Mean of column B" 109.4
" Difference between means (B - A) ± SEM" "0.5391 ± 13.95"
" 95% confidence interval" "-29.85 to 30.93"
" R squared (eta squared)" 0.0001244

"F test to compare variances"
" F, DFn, Dfd" "3.075, 6, 6"
" P value" 0.1974
" P value summary" ns
" Significantly different (P < 0.05)?" No

"Data analyzed"
" Sample size, column A" 7
" Sample size, column B" 7
