# Supplementary material for: Influence of fermented feed additive on gut morphology, immune status, and microbiota in broilers
Source: BMC Vet Res. 2022 Jun 10;18:218. doi: 10.1186/s12917-022-03322-4 (PMC9185985; doi:10.1186/s12917-022-03322-4)
Supplement: Supplementary file 1 — Additional file 1. [file 12917_2022_3322_MOESM1_ESM.zip › test of IntestineCD-1.pdf]

"Table Analyzed" Intestine (CD)

"Column C"	FFL
vs.	vs.
"Column B"	PC

"Unpaired t test"
"	P value"	0.5902
"	P value summary"	ns
"	Significantly different (P < 0.05)?"	No
"	One- or two-tailed P value?"	Two-tailed
"	t, df"	"t=0.5563, df=10"

"How big is the difference?"
"	Mean of column B"	122.3
"	Mean of column C"	118.6
"	Difference between means (C - B) ± SEM"	"-3.711 ± 6.670"
"	95% confidence interval"	"-18.57 to 11.15"
"	R squared (eta squared)"	0.03002

"F test to compare variances"
"	F, DFn, Dfd"	"9.924, 6, 4"
"	P value"	0.0437
"	P value summary"	*
"	Significantly different (P < 0.05)?"	Yes

"Data analyzed"
"	Sample size, column B"	7
"	Sample size, column C"	5
