# Supplementary material for: Influence of fermented feed additive on gut morphology, immune status, and microbiota in broilers
Source: BMC Vet Res. 2022 Jun 10;18:218. doi: 10.1186/s12917-022-03322-4 (PMC9185985; doi:10.1186/s12917-022-03322-4)
Supplement: Supplementary file 1 — Additional file 1. [file 12917_2022_3322_MOESM1_ESM.zip › test of VH-1.pdf]

"Table Analyzed" (VH)

"Column C" FFL
vs. vs.
"Column B" PC

"Unpaired t test"
" P value" 0.1748
" P value summary" ns
" Significantly different (P < 0.05)?" No
" One- or two-tailed P value?" Two-tailed
" t, df" "t=1.451, df=11"

"How big is the difference?"
" Mean of column B" 507.0
" Mean of column C" 663.8
" Difference between means (C - B) ± SEM" "156.9 ± 108.1"
" 95% confidence interval" "-81.13 to 394.9"
" R squared (eta squared)" 0.1606

"F test to compare variances"
" F, DFn, Dfd" "1.531, 6, 5"
" P value" 0.6567
" P value summary" ns
" Significantly different (P < 0.05)?" No

"Data analyzed"
" Sample size, column B" 7
" Sample size, column C" 6
