# Supplementary material for: Influence of fermented feed additive on gut morphology, immune status, and microbiota in broilers
Source: BMC Vet Res. 2022 Jun 10;18:218. doi: 10.1186/s12917-022-03322-4 (PMC9185985; doi:10.1186/s12917-022-03322-4)
Supplement: Supplementary file 1 — Additional file 1. [file 12917_2022_3322_MOESM1_ESM.zip › test of VH_CD-1.pdf]

```
"Table Analyzed"  (VH/CD)

"Column C"       FFH
vs.     vs.
"Column B"       PC

"Unpaired t test"
"    P value"    0.0683
"    P value summary"    ns
"    Significantly different (P < 0.05)?" No
"    One- or two-tailed P value?" Two-tailed
"    t, df"      "t=2.021, df=11"

"How big is the difference?"
"    Mean of column B"   4.524
"    Mean of column C"   6.105
"    Difference between means (C - B) ± SEM"       "1.581 ± 0.7822"
"    95% confidence interval"     "-0.1405 to 3.303"
"    R squared (eta squared)"     0.2708

"F test to compare variances"
"    F, DFn, Dfd"        "1.229, 5, 6"
"    P value"    0.7970
"    P value summary"    ns
"    Significantly different (P < 0.05)?" No

"Data analyzed"
"    Sample size, column B"       7
"    Sample size, column C"       6
```
