# Supplementary material for: Influence of fermented feed additive on gut morphology, immune status, and microbiota in broilers
Source: BMC Vet Res. 2022 Jun 10;18:218. doi: 10.1186/s12917-022-03322-4 (PMC9185985; doi:10.1186/s12917-022-03322-4)
Supplement: Supplementary file 1 — Additional file 1. [file 12917_2022_3322_MOESM1_ESM.zip › test VH_CD.pdf]

"Table Analyzed" (VH/CD)

"Column D" FFH
vs. vs.
"Column B" PC

"Unpaired t test"
" P value" 0.0327
" P value summary" *
" Significantly different (P < 0.05)?" Yes
" One- or two-tailed P value?" Two-tailed
" t, df" "t=2.476, df=10"

"How big is the difference?"
" Mean of column B" 5.886
" Mean of column D" 7.099
" Difference between means (D - B) ± SEM" "1.212 ± 0.4896"
" 95% confidence interval" "0.1215 to 2.303"
" R squared (eta squared)" 0.3801

"F test to compare variances"
" F, DFn, Dfd" "1.100, 6, 4"
" P value" 0.9703
" P value summary" ns
" Significantly different (P < 0.05)?" No

"Data analyzed"
" Sample size, column B" 7
" Sample size, column D" 5
