# Supplementary material for: Influence of fermented feed additive on gut morphology, immune status, and microbiota in broilers
Source: BMC Vet Res. 2022 Jun 10;18:218. doi: 10.1186/s12917-022-03322-4 (PMC9185985; doi:10.1186/s12917-022-03322-4)
Supplement: Supplementary file 1 — Additional file 1. [file 12917_2022_3322_MOESM1_ESM.zip › test of VH.pdf]

"Table Analyzed" (VH)

"Column B" NC
vs. vs.
"Column A" PC

"Unpaired t test"
" P value" 0.3951
" P value summary" ns
" Significantly different (P < 0.05)?" No
" One- or two-tailed P value?" Two-tailed
" t, df" "t=0.8820, df=12"

"How big is the difference?"
" Mean of column A" 593.1
" Mean of column B" 507.0
" Difference between means (B - A) ± SEM" "-86.16 ± 97.69"
" 95% confidence interval" "-299.0 to 126.7"
" R squared (eta squared)" 0.06088

"F test to compare variances"
" F, DFn, Dfd" "2.042, 6, 6"
" P value" 0.4061
" P value summary" ns
" Significantly different (P < 0.05)?" No

"Data analyzed"
" Sample size, column A" 7
" Sample size, column B" 7
